# Combining environmental and socioeconomic data to understand determinants of conflicts in Colombia

**DOI:** 10.3389/fdata.2023.1107785

**Published:** 2023-02-16

**Authors:** Stefania Fiandrino, Ciro Cattuto, Daniela Paolotti, Rossano Schifanella

**Affiliations:** ^1^Institute for Scientific Interchange, ISI Foundation, Turin, Italy; ^2^Department of Computer Science, University of Turin, Turin, Italy

**Keywords:** data science, social impact, conflicts, Colombia, spatial analysis, human rights, Sustainable Development Goals

## Abstract

Conflicts cause immense human suffering, violate human rights, and affect people's stability. Colombia is affected for decades by a high level of armed conflicts and violence. The political and socio-economic situation, drug trafficking in the Colombian economy, and natural disasters events affect the country and foster general violence. In this work, we aim to evaluate the role of the socioeconomic, political, financial, and environmental determinants of conflicts in the Colombian context. To achieve these aims, we apply a spatial analysis to explore patterns and identify areas that suffer from high levels of conflict. We investigate the role of determinants and their relationship with conflicts through spatial regression models. In this study, we do not consider only the entire Colombian territory, but we extend the analysis to a restricted area (Norte de Santander department) to explore the phenomena locally. Our findings indicate a possible diffusion process of conflicts and the presence of spillover effects among regions by comparing the two most known spatial regression models. As regards possible key drivers of conflicts, our results show that surprisingly socioeconomic variables present very little relationship with conflicts, while natural disasters and cocaine areas show a relevant impact on them. Despite some variables seeming to be the more informative to explain the process globally, they highlight a strong relationship for only a few specific areas while considering a local analysis. This result proves the importance of moving to a local investigation to strengthen our understanding and bring out additional interesting information. Our work emphasizes how the identification of key drivers of violence is crucial to have evidence to inform subnational governments and to support the decision-making policies that could assess targeted policy options.

## 1. Introduction

Colombia has suffered from decades of internal conflicts, one of the longest non-international armed conflicts. Nearly 9 million people are registered as victims of armed conflicts since 1985 (source Unidad para las Víctimas, UARIV), 8 million internally displaced people since 1985 (source UARIV), 8.5 million people in need (United Nations Office for the Coordination of Humanitarian Affairs, UNOCHA) (Council, 2021). In 2016, the Government of Colombia signed a peace agreement with the countrys largest guerrilla group, the FARC. However, several other armed groups remain active across the country and civilians continue to suffer the humanitarian consequences of the ongoing conflicts and other forms of violence. The International Committee of the Red Cross has recently defined the armed conflict in Colombia as a pain that does not go away (International Committee of the Red Cross, 2021). In this context, the recognition of the historical situation in which Colombia finds itself turns out to be crucial. This is the first step for taking decisive actions that could help in reducing or stopping violence and in guarantying human rights in the whole country.

The socioeconomic instability creates suffering among the most vulnerable strata of the population even more from the lack of protection, food assistance, health care, education, and safe water. Historically, socioeconomic relationships in Colombia have created an unequal social and economic structure (Vásquez, 2011), and researchers considered inequality as a factor that strongly influences violence. According to Roncancio et al. (2020) a regionalization of vulnerability exists within the territory between areas of urban concentration where the industrial and economic growth is focused, and those rural areas of poor socioeconomic development. The last ones may also be marked by the presence of political conflict and the production of illegal crops. Conflict and drugs are connected and, in particular, the access by belligerents to the illicit economy strengthens the insurgent movements. The advantages that the insurgents gain from access to drug production and distribution include enlarged financial assets and improved military capabilities, an expansion of strategic and tactical options, and, crucially, an improvement in relations with local populations. The presence of large cocaine cultivation areas could be an indication of a place targeted by the belligerents, or even already controlled by them (Felbab-Brown, 2005). The drug issue is directly related to the socio-political and financial domain. In addition, the presence of large cocaine areas near the forest is linked to Colombian deforestation that impacts the environment (Negret et al., 2019). In this context, cocaine cultivation is recognized as playing an essential role in driving terrorism and violence. Vásquez (2011) suggests that the continuous struggle for land tenure to control coca cultivation is one of the main roots of the Colombian conflict. In addition to cocaine cultivation, coffee crops are particularly important: Colombia is the third largest coffee producing country in the world. Researchers focus on coffee as the largest Colombian exports to study whether there is some relationship with increase of violence. In particular, Dube and Vargas cosider the intensity of coffee crops in Colombian municipalitites and the national coffee price shocks. They state that the coffee price shocks have a negative relationship with conflict: when the price of coffee decreases, violence increase differentially in municipalities that produce coffee more intensively (Dube and Vargas, 2013). Thus, coffee areas could experience more an increase in violence than non-coffee areas due to price shocks. Also the issue of phenomena of natural origin and the disasters that they cause is of great interest because Colombia is positioned as one of the countries with the highest risk of natural disasters in the world, being classified as one of the countries with the highest mortality due to these events (Wilches et al., 2020). And vice versa, armed conflict in Colombia has significantly reconfigured natural rural landscapes and ecosystems through altered modes of subsistence, modes of economic production, and land cover (Corrales and Gutierrez, 2019). Recent studies suggest taking into account climate change that impacts the environment causing human displacement and social conflicts (Olagunju et al., 2021). Climate change increases natural disasters, drought, desertification and resource scarcity (Chilaka and Odoh, 2012). These situations would, in turn, result in loss of means of subsistence, and, consequently, displacement of people, migration in search of greener pasture, herdsmen migration and increased risks of armed conflict (Olagunju et al., 2021).

The goal of this work is to build a comprehensive data-driven framework to study the socio-economic and environmental determinants of violent internal conflicts in the Colombian context. Efforts to understand conflict dynamics are crucial to identify areas that continue to suffer from high levels of conflict and represent a key to meeting the Sustainable Development Goals (SDGs). In fact, armed conflict not only obstructs progress toward SDG-16 (peace, justice, and strong institutions) but also hampers SDG-3 (good health and well-being). Evaluating the role of socioeconomic and environmental determinants associated with conflicts and exploring the related relationship strengthen our understanding of the role played by both socioeconomic and environmental factors and our knowledge of the very complex system of conflicts phenomena.

In particular, in this work we address the following research questions: 1) by exploring the link among environment/climate variables, socioeconomic and conflict data, is there a certain set of circumstances for conflict to trigger? And how much do these variables influence conflicts? 2) is it possible to identify pockets of vulnerability/conflict areas using historical data?

To achieve these aims, we search for available open data that covers the domains we plan to take into account in the study (conflicts, environmental, socioeconomic). Firstly, we explore the presence of spatial patterns to identify areas that suffer from high levels of conflict. Then, starting from a vast set of control variables potentially useful to investigate the conflicts phenomena, we apply a methodology of dimensionality reduction to obtain the most significant determinants. We compare two spatial regression models to study which variable is statistically significant and positively or negatively related to conflicts. We extend the analysis by considering also information about past conflicts, by moving to a local spatial regression model that allows exploring the phenomena locally, and by selecting a department to narrow down the research on a restricted area and explore how relationships change. A related research body (Holmes et al., 2018) has focused on mapping spatial and spatiotemporal conflict patterns and estimating vulnerability caused by violent events by evaluating the role of socioeconomic and environmental determinants. Our analysis extends these studies in several directions: 1) we include the dimension of natural phenomena impacting the environment; 2) we focus on local rather than national spatial relationships; 3) we base the research on more recent human rights violations data (data we used comes from OCHA, The Coordination of Humanitarian Affairs).

## 2. Materials and methods

### 2.1. Study area and data

The analysis covers the entire Colombian territory and it is performed at a municipality level. We consider the 1,122 municipalities because the smallest Colombian administrative unit facilitates the modeling of phenomena at a local scale. Due to data availability at the municipal spatial granularity, we initially focus our analysis on 2018.

The conflict and violence status in Colombia is represented by a combination of violations of humanitarian norms, the absence of the State in many areas of the country, and the presence of armed groups that commit abuses against civilians. The International Committee of Red Cross states that in 2018 an increase in mass displacement, in antipersonnel mines and explosive devices has been recorded. This trend is compounded by a list of abuses such as homicides, threats, and disappearances. Some of them are committed by gangs that exert social control and perpetrate various forms of armed violence in urban neighborhoods and their peripheries (ICRC, 2018). Starting from this report, we search for public data that takes into account these violent and conflict events. The United Nations Office for Coordination of Human Affairs (OCHA) provides data about violent events that occurred in Colombia. This situational monitor is a tool that allows to visualize on a map which is the most affected places in Colombia for both conflicts and natural disasters, to have a summary of the situation at a country level (e.g., number of acts of violence, number of natural disasters events, number of victims by population groups, ...) and to retrieve data related to the violent events and disasters events occurred per year. This dataset is event based, and includes nearly 3000 episodes in over 400 Colombian municipalities in 2018. We extract information about the date on which the events happened, the event type, and the admin1, admin2, and admin3 levels. The dataset covers different event types: armed actions, attacks on military or police infrastructure, illegal roadblocks and/or checkpoints, fighting, ambush, clash of non-state actors, fire events, harassment, raids, attack on illicit war targets, intermediate mass displacement, anti-personnel mines and explosive devices, sexual violence, homicides, threats (individual/collective), enforced disappearance, kidnapping, hostage taking, torture. In the Supplementary material we have included some summary statistics on conflict events. We report the distribution of conflicts at department level and the monthly time series of conficts in Colombia in 2018 at department level. We highlight Norte the Santander, Narino and Antioquia as the most affected departments in 2018. Also, we observe heterogeneity among departments in terms of periods with increased or decreased conflicts. Regarding the independent demographic, socioeconomic and environmental variables, we start with a set of variables based on a discussion with local experts and we relied on open datasets described in the following. TerriData is a tool created by the Colombian National Planning Department (DNP) and it provides data for monitoring, analyzing, and strengthening public management based on Colombia's municipal, provincial and regional statistics. The platform collects information about demography, access to public services, education, health, finance, economy, territorial planning, job market, and environment. To include data about cocaine cultivation areas and coffee cultivation areas, we rely on open data collected in the Datos Abiertos portal. In particular, from the dataset related to cocaine cultivation areas we extract the total percentage of cocaine area per municipality, while from the dataset related to coffee owned by the Department of Agriculture, we extract data on harvested areas, sown areas, production and yield per municipality. Finally, to add more detailed information about demography not available from TerriData (gender structure and age structure) we manipulate the National Population and Housing Census 2018 available in the National Administrative Department of Statistics (DANE).

In the second stage of the analysis, we also analyzed historical data about conflicts to explore whether and how much information of the previous years influence the intensity of violent events in the current year. Data are prepared and preprocessed with the *pandas* library in *Python*.

### 2.2. Methods

#### 2.2.1. Spatial units and exploration

A study related to leftist guerrilla violence illustrates the importance of moving to a sub-national analysis (Holmes et al., 2018). Researchers found out that although broad regions or departments capture some of the variations, there are different patterns at the municipal level and there are also different degrees of clustering at the department or the municipal level. Thus, we decide to perform the research at a municipality level, focusing on conflicts that occurred in 2018. To explore the country's situation in terms of conflict intensity, we visualize on a map where conflicts take place. This allows for showing areas that suffer from a high concentration of conflicts and capturing spatial patterns. For the analysis, we use the *pysal* library in *Python*.

#### 2.2.2. Spatial analysis

In the context of this study, Exploratory Spatial Data Analysis (ESDA) is used for the analysis of the spatial autocorrelation of conflicts. The objective is to statistically determine whether similar values in the neighboring regions were likely to occur. The global Moran index measures the spatial correlation of geographic locations using a given variable (conflicts). We test the hypothesis of nonzero spatial autocorrelation in violent events by computing the Local Morans I statistic. Thus, we evaluate municipal spatial patterns. Spatial dependence reflects a situation where values observed at one location or region depend on the values of neighboring observations at nearby locations (Okunlola et al., 2021). The visual inspection of the map pattern for the violent events is the first step to search for spatial structure. We can recognize darker clusters and thus, areas with a little concentration of violent events, and lighter areas with a higher intensity of conflicts. If the spatial distribution of conflicts is random, we should not see any clusters on the map. However, our visual system may have detected no statistical patterns. The Morans I statistic is used to representing the clustering degree to characterize the global spatial patterns of the variable in this study (Anselin, 1995; Al-Ahmadi and Al-Zahrani, 2013). The Moran's *I*-value is expressed as:


(1) $$I=\frac{n\sum_{i}W_{i,j}(X_{i}-\bar{X})(X_{j}-\bar{X})}{\sum_{i}\sum_{j}W_{i,j}\sum_{i}{\left(X_{i}-\bar{X}\right)}^{2}}$$

where *X*_*i*_ is the number of conflicts at position *i* in a municipality, $\bar{X}$ is the averaged number of conflicts for the Colombian municipalities, *X*_*j*_ is the number of conflicts at position *j* in the municipality, *W*_*i, j*_ is the neighborhood matrix for the municipalities *i* and *j*, which represent proximity, and *n* is the number of municipalities.

The concept of spatial autocorrelation relates to the combination of two types of similarity: spatial similarity and attribute similarity. The measure of spatial autocorrelation combines these two types of similarity into a summary measure. In spatial autocorrelation analysis, the spatial weights are used to formalize the notion of spatial similarity. According to Anselin et al. (2007), each element *W*_*i, j*_ of the neighborhood matrix *W* represents a proximity measurement between the municipalities (polygons) *i* and *j*, which can be calculated from one of several criteria (distance between the centroid, contiguity (queen), nearest neighbors). In this study, we use the k-nearest neighborhood in which we create the nearest neighbor weights matrix based on k nearest neighbors. Thus, the spatial weight between neighborhoods *i* and *j* indicates if the two are neighbors (i.e., geographically similar). What we also need is a measure of attribute similarity to pair up with this concept of spatial similarity. The spatial lag is a derived variable that accomplishes this. For neighborhood, the spatial lag is defined as:


(2) $$\begin{array}{l} ylag_{i}=\underset{j=0}{\sum }w_{i,j}y_{j} \end{array}$$

We turn to formal statistical measures of spatial autocorrelation to complement the visualization. In particular, we examine the spatial dependence by computing Moran's I statistic. Although the global spatial correlation tests identify general trends in all 1,101 municipalities, it is also necessary to know which areas have a higher and lower spatial correlation. The LISA method quantifies the presence of spatial correlation or clustering. The method identifies which municipalities in the study have similar characteristics (Anselin, 1995). Taking that into account, the local Morans I is calculated in all Colombian municipalities.

#### 2.2.3. Analysis of determinants

In order to explore the potential environmental and socioeconomic factors associated with conflicts, we implement a pipeline that involves feature normalization and selection steps. To avoid redundancy within the model we check for multicollinearity and if we observe strong correlations among a group of variables (ρ > 0.8) we filter out the ones with less correlation with the target. To reduce model complexity we implement the *Forward Stepwise* selection strategy in which at the beginning the model contains no predictors and then, at each step, the variable that gives the greatest additional improvement to the fit is added to the model. In order to analyze whether the variable selection method suffers from model instability, we study the stability of random perturbations of training samples using the methodology proposed in Sauerbrei et al. (2015). We implement a subsampling that randomly selects 63.2% of the initial dataset, and we run the selection procedure on the subsample, considering the Akaike's Information Criteria (AIC) minimum value. We select this threshold so that the number of observations is, on average, the same as the number of unique observations in a bootstrap pseudo-sample. The subsampling technique has been extensively studied, and it shows asymptotic consistency even in cases where the classical bootstrap fails (Chernick, 2011; Schifanella et al., 2020). We perform 300 subsampling iterations and each time we extract the subset of features that shows the minimum AIC value. We compute the stability estimator proposed by Nogueira et al. (2017) that proves an intermediate to good stability. The stability value is 0.51 (confidence intervals: lower = 0.50, upper = 0.53). We select the subset that occurs the highest number of times. Several features get filtered out because of multicollinearity issues, such as demographic variables related to gender and age structure, total income, policy-related variables (investment in social promotion, community development, education, health...). In the second stage of the feature selection approach, also variables related to coffee cultivation, other demographic data such as the number of people belonging to ethnic minorities, some environmental and social variables are excluded from the final set of features. The final set of covariates is reported in Table 1. We also analyzed the outcome of two additional feature selection approaches on the variables final set: the VIF method and the Random Forest method based on Gini. More information about our feature selection method and the sensitivity analysis is reported in the Supplementary material to highlight that different feature selection approaches eventually produce similar results. For the analysis, we use the *scikit-learn* module in *Python*.

**Table 1:** Explanatory variables considered after *feature selection* step.

**Variable**	**Type**	**Source**
**Demography**		
Indigenous population	People	TerriData, with Departamento Administrativo Nacional de Estadística (DANE) data from the National Population and Housing Census
**Education**		
Net coverage in basic education	%	TerriData, with DANE data
Rural Illiteracy Rate (Census)	%	TerriData, with DANE data
**Environment**		
Disasters Events 2017	%	OCHA
**Finance**		
Investment - Agriculture	Millions of current pesos	TerriData, with data from FUT
**Access to public services**		
Electric Power Coverage (Census)	%	TerriData, with DANE data
Natural Gas Coverage (Census)	%	TerriData, with DANE data
Sewer Coverage (Census)	%	TerriData, with DANE data
**Health**		
Mortality Rate	Cases for 1,000 inhabitants	TerriData, with Ministry of Health and social protection data (MSPS)
Cocaine cultivation areas	% per municipality	GOVCO portal

#### 2.2.4. Global spatial regression models

Once we have selected the most suitable subset of features, we compare the two most-known global spatial regression models, the Error Model and the Lag Model.

**Figure 1:**
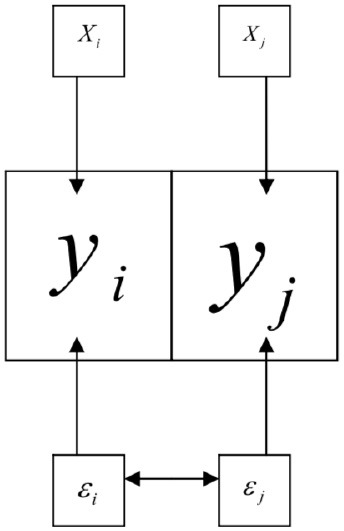
The figure shows the scheme of the spatial error model (Catma, 2021).

**Figure 2:**
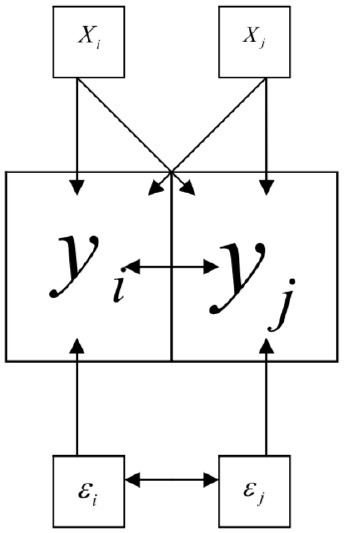
The figure shows the scheme of the spatial lag model (Catma, 2021).

The Spatial Error Model (Anselin, 1980) is represented by the following expressions:


$$y=X\beta +\lambda Wu+\epsilon \;\text{,}\;\epsilon ∽N\left(O,\sigma^{2}I_{n}\right)$$

where λ in these expressions is the spatial autoregression parameter of error term *u*. This model includes the spatial autoregression of the error term in the normal multiple regression model; that is, this examines whether the error term has a spatial dependency (Figure 1). The Spatial Lag Model includes a spatially lagged dependent variable. Formally, the model is represented by the following expression:


$$y=\rho Wy+X\left(\beta \right)+\epsilon \;\text{,}\;\epsilon ∽N\left(O,\sigma^{2}I_{n}\right)$$

where *Wy* is the spatially lagged dependent variable for weights matrix *W*; *X* is the matrix of observations on the explanatory variable; ϵ is the vector of error terms; ρ is the coefficient of spatial lag that measures the intensity of the interdependencies of the neighboring conflicts on the number of conflicts of each municipality and β is a parameter. In the Spatial Lag Model the dependent variable *y* in place *i* is affected by the independent variables in both place *i* and *j* (Figure 2).

We compare the two types of spatial regression models using a Maximum Likelihood approach.

#### 2.2.5. Local spatial regression models

Global spatial regression models assume homogeneity throughout the data. Specifically, global models assume that the same stimulus provokes the same response in all parts of the study region. However, this assumption turns out to be restrictive because, in practice, the relationships between variables might be non-stationary and vary geographically (Cressie, 1993; Jones III and Hanham, 1995). If nonstationarity exists then there is a suggestion that different processes are at work within the study region. Therefore, we apply the Geographically Weighted Regression Model (GWR) to understand the phenomena locally, under the assumption of spatial heterogeneity. According to Goldstein (2020), because the assumptions put into the GWR model have proven to be rather sensitive, we use it as an exploratory tool to integrate our analysis. GWR is a local regression technique that is used to measure how the strength of the relationships among the dependent and explanatory variables differ from location to location (Delmelle et al., 2016). The conventional regression equation can be expressed as:


(3) $$\begin{array}{l} \hat{y_{i}}=\beta_{0}+\underset{k}{\sum }\beta_{k}x_{i,k}+\epsilon_{i} \end{array}$$

where $\hat{y_{i}}$ is the estimated value of the dependent variable for observation *i*, β_0_ is the intercept, β_*k*_ is the parameter estimate for variable *k*, *x*_*i, k*_ is the value of the *k*^*th*^ variable for *i*, and ϵ_*i*_ is the error term. Instead of calibrating a single regression equation, GWR generates a separate regression equation for each observation. Each equation is calibrated using a different weighting of the observations contained in the dataset. Each GWR equation may be expressed as:


(4) $$\begin{array}{l} \hat{y_{i}}=\beta_{0}\left(u_{i},v_{i}\right)+\underset{k}{\sum }\beta_{k}\left(u_{i},v_{i}\right)x_{i,k}+\epsilon_{i} \end{array}$$

where (*u*_*i*_, *v*_*i*_) captures the coordinate location of *i* (Fotheringham et al., 1998). The assumption is that observations nearby one another have a greater influence on one anothers parameter estimates than observations farther apart. The weight assigned to each observation is based on a distance decay function centered on observation *i* and the distance between observations is calculated as the distance between polygon centroids.

## 3. Results

### 3.1. Spatial analysis

**Figure 3:**
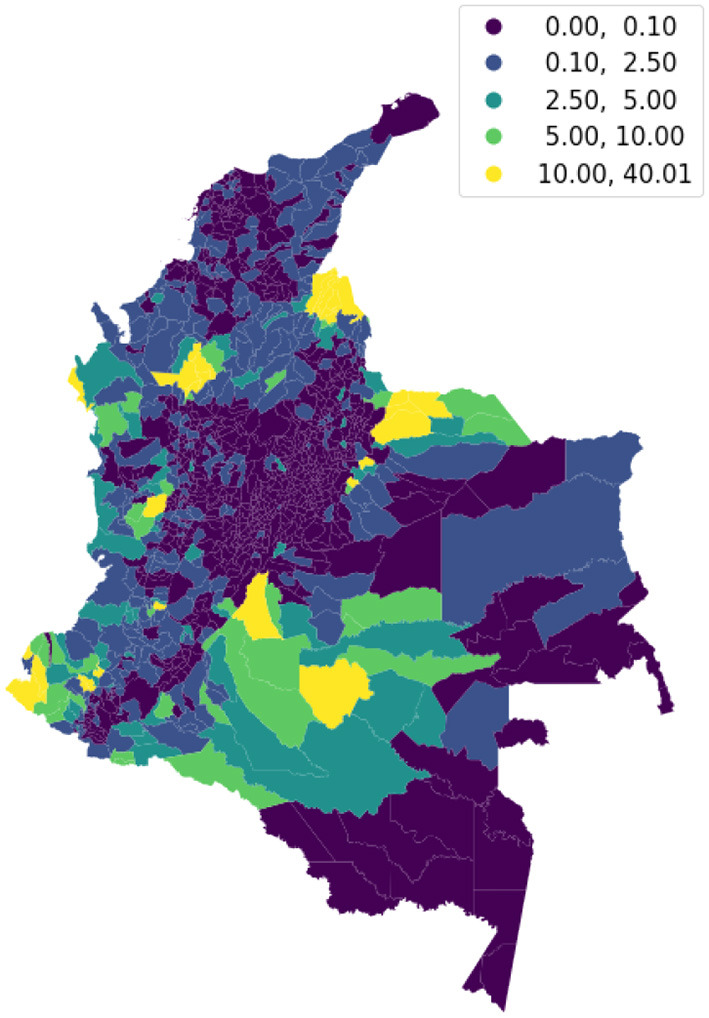
Distribution of violent events in 2018, per 10,000 inhabitants, by the municipality.

**Figure 4:**
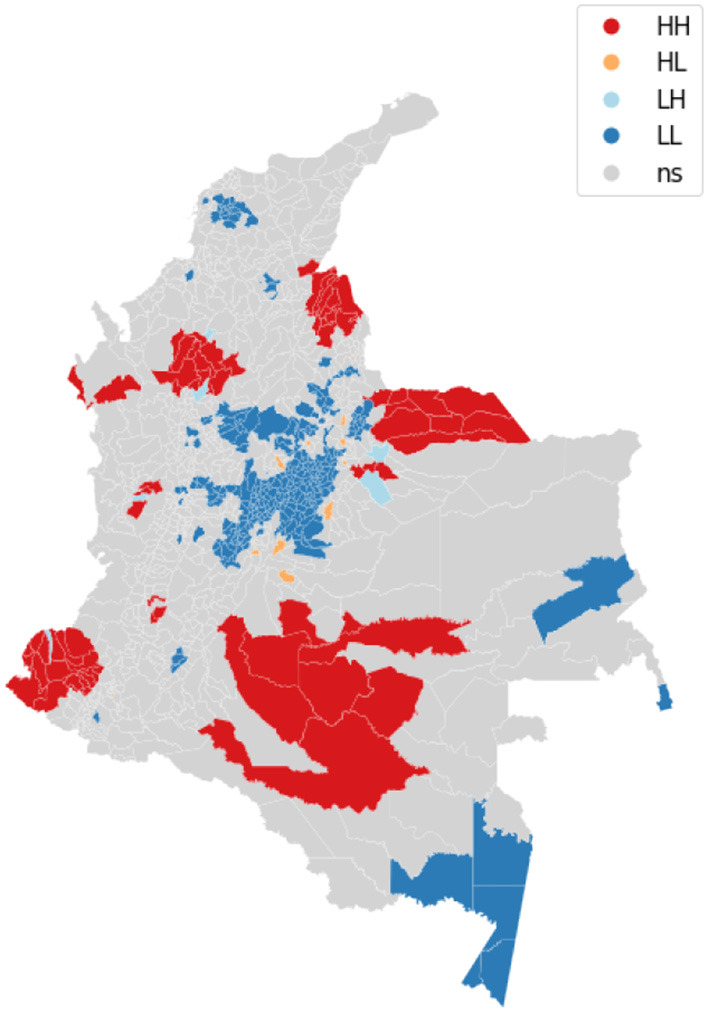
The figure shows the LISA clusters map.

**Figure 5:**
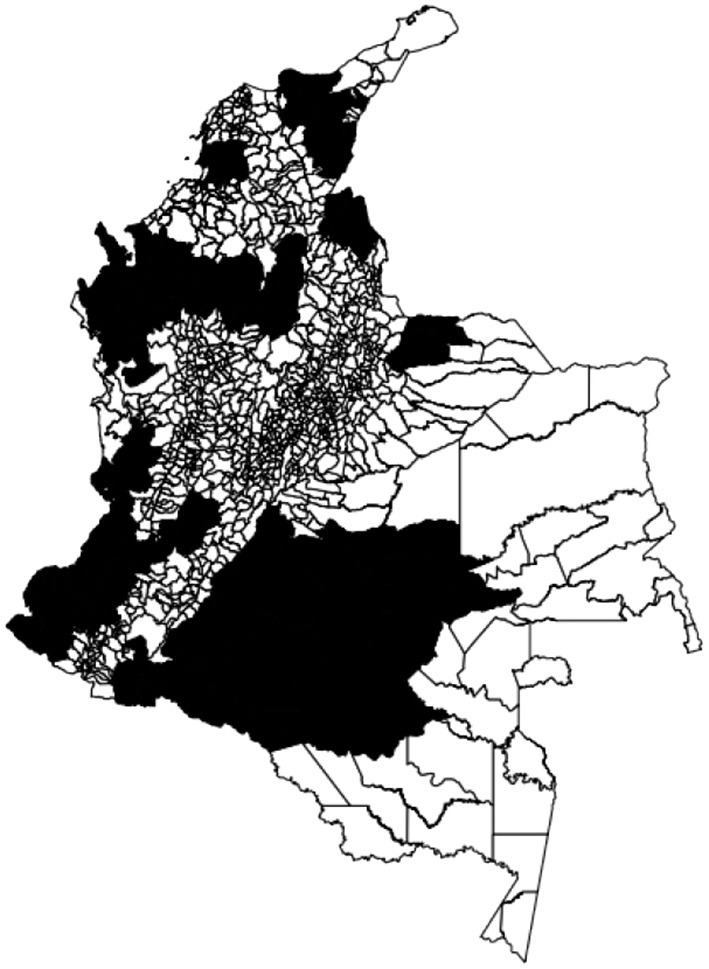
The figure shows the PDET regions (in black).

We visualize on a map the intensity of conflicts per municipality to evaluate the presence of spatial dependence and patterns. Figure 3 shows the map of the target variable. Then, we apply the Global Moran's I statistic to test the spatial autocorrelation of the conflict-related process. The interpretation of results is done within the context of the null hypothesis that the analyzed attribute is randomly distributed in the study area. The Moran's *I*-value is 0.4, the *p*-value (0.001) is statistically significant, and the z-score (30.06) is positive, thus we can reject the null hypothesis. The spatial distribution of high values and/or low values in the dataset is more spatially clustered than would be expected if the spatial processes are random. We accept the alternative hypothesis of clustering which is a characteristic of the complete spatial pattern and does not provide an indication of the location of the clusters. In order to highlight which areas show the higher or lower spatial correlation, we perform the local spatial autocorrelation and distinguish the specific types of local spatial autocorrelation in High-High, Low-Low, High-Low, and Low-High. These types of local spatial autocorrelation describe similarities or dissimilarities between a specific polygon with its neighboring polygons. Then, we proceed with the Local Indicator of Spatial Association (LISA). This analysis provides a statistic for each location with an assessment of significance and establishes a proportional relationship between the sum of the local statistics and a corresponding global statistic. We are able to visualize on a map (Figure 4) the significant areas with a *p*-value lower than 0.05. We compare significant areas characterized by the label HH and so, a high number of conflicts, with the PDET regions. PDET stands for 'Planes de Desarrollo con Enfoque Territorial' (Development Plans with Territorial Approach). There are 16 PDET regions covering 170 municipalities. The PDETs were created in 2016 after the signing of the Peace Agreement with FARC. These plans are pianification tools intended to stabilize and transform the municipalities most affected by the internal conflict. Figure 5 shows the municipalities that belong to the PDET region's list. We can observe that areas marked in 2016 as the most suffering ones are still the most hit by conflicts in 2018.

### 3.2. Spatial regression models

**Figure 6:**
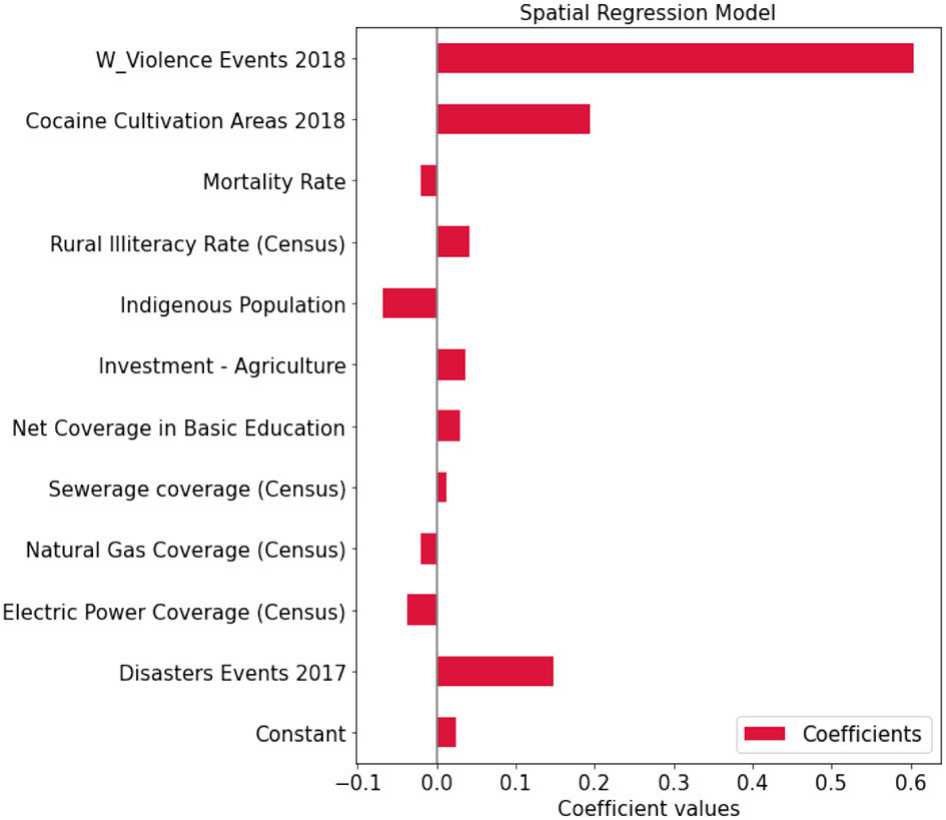
Regression coefficients—analysis on the entire colombian territory.

**Figure 7:**
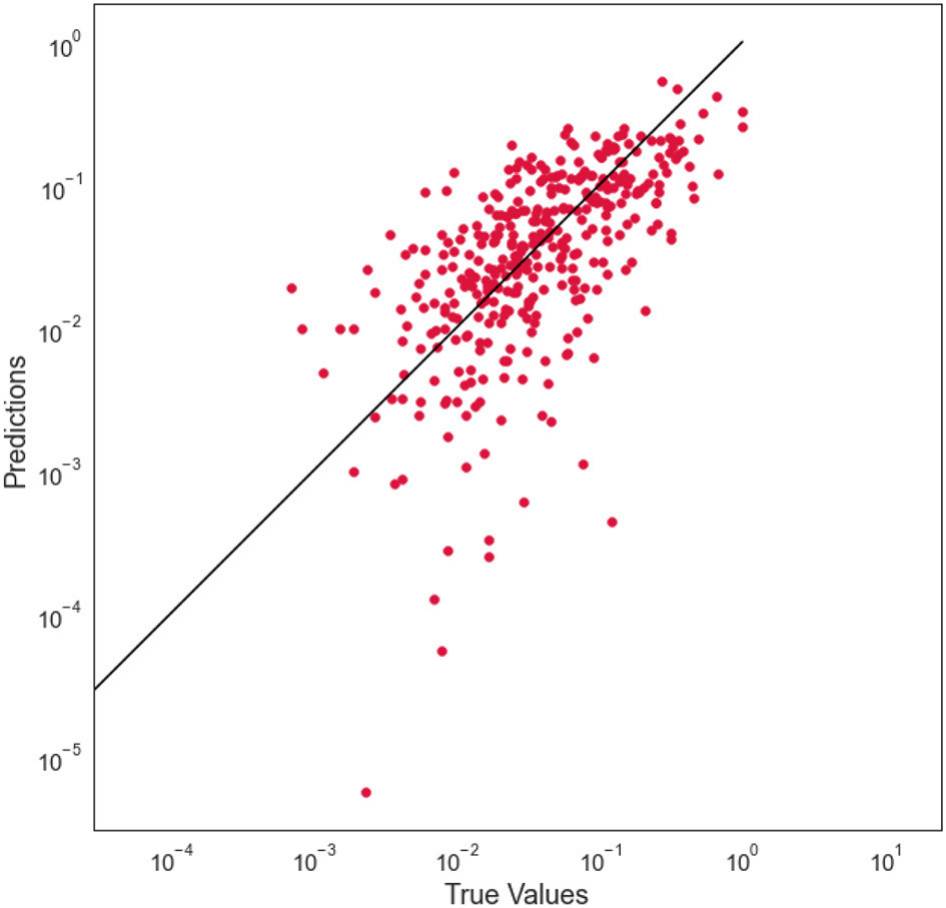
Actual vs. predicted—spatial lag model.

For model selection, Anselin et al. (2008) provide Lagrange Multiplier tests (LM tests) for a spatially lagged dependent variable and for a spatial error term under panel data setting (Anselin et al., 2008). Elhorst (2010) also suggested the robust counterparts of the LM tests. Table 2 reports the LM test statistics and the robust LM test statistics and the associated *p*-values to determine which spatial terms are appropriate. This is the so-called specific-to-general approach that tests the non-spatial model against the spatial lag and/or the spatial error model (Elhorst, 2014). We can see both simple tests of the lag and error are significant, indicating the presence of spatial dependence. To understand what type of spatial dependence may be at work we move to the robust tests. Again, both tests are significant, so we consider the largest value to guide the model selection. In the context of this study, the LM tests point out the lag model as more suitable than the error model to explain the conflicts phenomena system. To support the preliminary test, we compare the two models and explore the relationship between the prediction error at each site and the prediction error at the site nearest to it. We find out that the error model tends to cluster more than the lag model and thus when the model tends to over-predict conflicts in a location, sites around are more likely to also be over-predicted. In detail, Moran's *I*-values on residual are, respectively for the lag model and the error model, 0.012 (no spatial autocorrelation on the residuals) and 0.26. We notice that the lag model is the more appropriate and Figure 6 reports the coefficients of the run. Among the ten indicators, considering a *p*-value lower than 0.05, cocaine cultivation areas per municipality, natural disasters in 2017, rural illiteracy rate, and sewer coverage are positively related to conflicts; while electric power coverage and natural gas coverage are negatively related to conflicts; and indigenous population, investments in agriculture, mortality rate, net coverage in basic education and sewerage coverage seem to do not play relevant role. Besides this information, the spatial lag term of conflicts appears as an additional indicator (W Violence Events 2018). Its coefficients reflect the spatial dependence inherent in the sample data, measuring the average influence on observations by their neighboring observations. It has a positive effect and it is highly significant. Finally, Figure 7 shows the relationship between true values and predicted values.

**Table 2:** Lagrange multiplier tests (the numbers in parentheses are *p*-values).

	**Spatial lag** **(H0 : ρ = 0)**	**Spatial error** **(H0 : λ = 0)**
LM	294.46 (0.000)	265.42 (0.000)
Robust LM	37.65 (0.000)	8.60 (0.0034)

Analysis of the entire Colombian territory.

### 3.3. Exploring past conflicts contribution

In the first stage of the analysis, we focus on environmental and socioeconomic data to explain conflict phenomena. Then, we consider the autoregressive component to check whether and how much it improves the prediction. We base the analysis on the following hypothesis: past conflicts in a place could cause vulnerability and increase the likelihood of future conflicts in the same place. Thus, starting from OCHA violence events data, registered for the period 2008–2021, we perform a correlation analysis to highlight whether the number of conflicts in year *i* is correlated with the number of conflicts in year *j*. We find that by involving the data from all past years we would run into multicollinearity problems. For this reason, we filter out all the variables that have a correlation coefficient higher than 0.8 among each other and we select the feature that shows higher correlation with the outcome variable (conflicts in 2018). The selected variable is the number of conflicts that have occured in the previous year, 2017, that proves a strong correlation (Pearson coefficient 0.93). In the Supplementary material we report the anlaysis. This process allows to avoid redundancy in the model, to include information about the past situation and to explore the autoregressive component of the problem. We compare the autoregressive model and the hybrid model that contains information covering the three domains: historical conflicts data (2017), environmental features, and social information. We notice that the autoregressive model is the baseline (*Pseudo R-squared* = 0.58 and *Spatial Pseudo R-squared* = 0.47). However, adding exogenous variables the overall predictive power of the model increases (*Pseudo R-squared* = 0.61 and *Spatial Pseudo R-squared* = 0.55). The summary of output shows that the indicator regarding past events is positively related to current violence events and its regression coefficient (0.63) is highly significant. On the other hand, we notice that the most of the exogenous variables do not play relevant role and only disasters events in 2017, electric power coverage, net coverage in basic education, and cocaine cultivation areas show coefficients with significant *p*-values.

### 3.4. Geographically weighted regression model

**Figure 8:**
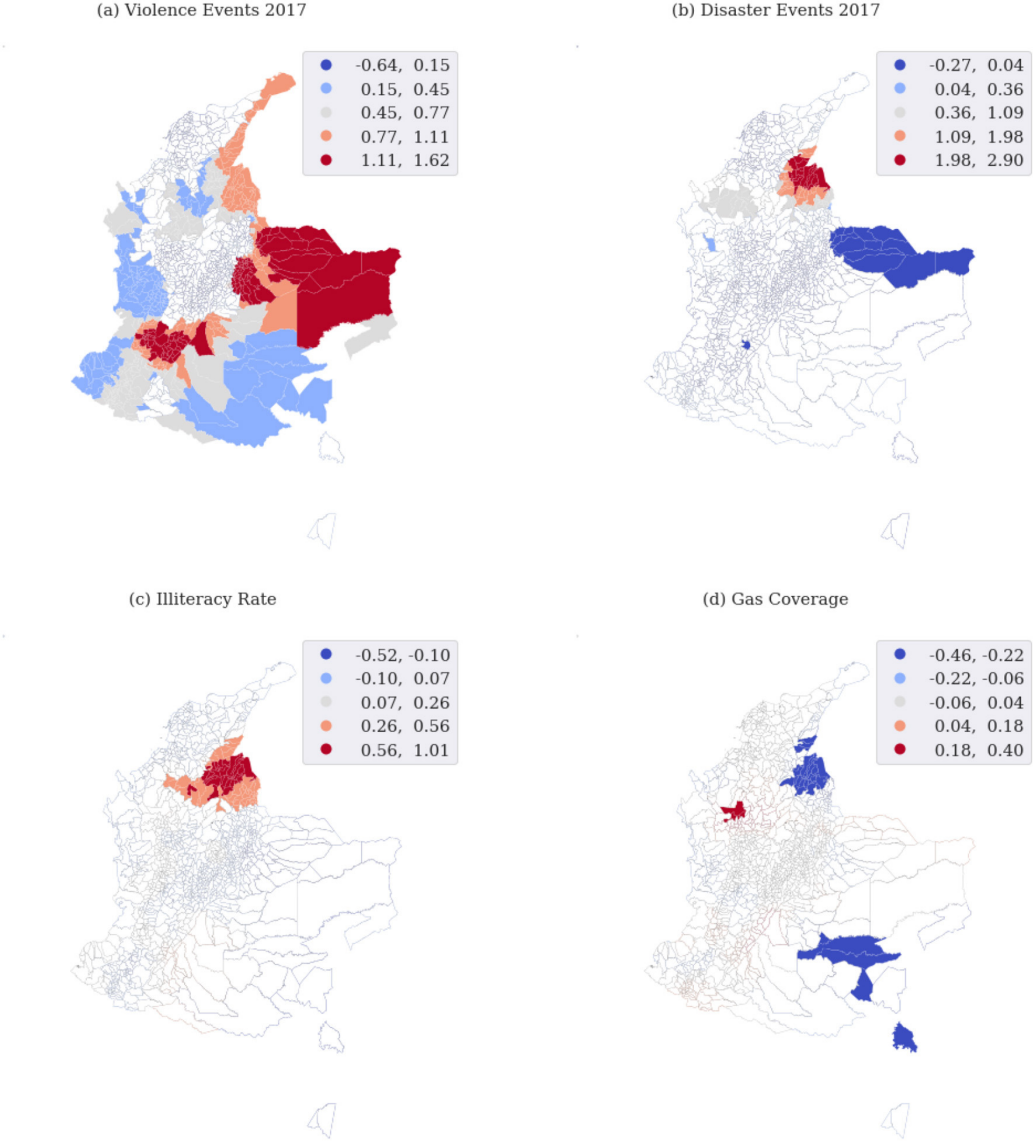
Geographically weighted regression model results: the maps show areas where the local parameters have significant local *t*-values and significant *p*-values.

We apply the GWR considering the same predicted variables used for the global models. We map GWR results because the mapping facilitates interpretation based on spatial context and known characteristics of the study area (Goodchild and Janelle, 2004). According to Matthews and Yang (2012), mapping only the parameter estimate alone is misleading, as the map reader has no way of knowing whether the local parameter estimates are significant anywhere on the map. For this reason, we extract the separate parameter, *t*-value, that identifies the goodness-of-fit and is calculated for each observation. These values can thus be mapped, allowing the analyst to visually interpret the spatial distribution of the nature and strength of the relationships among explanatory and dependent variables (Mennis, 2006). Starting from these points, we provide the maps with adequate information in order to be able to discern the areas where local parameter estimates have significant local *t*-values. We report the most interesting maps to be discussed, which cover all aspects and domains taken into account in the study. Analyzing the map of violent events in 2017 (Figure 8a), we can observe several significant areas that show the expected positive relationship between historical events and conflicts in 2018. On the other hand, by mapping environmental and socio-economic coefficients we have seen that only a few areas are significant. Figure 8b is related to the relationship between disaster events in 2017 and conflicts, under the hypothesis that natural disasters may force migration and will increase vulnerability. We find out that the expected positive relationship between natural disasters and conflicts is concentrated in very few areas of the following departments: Norte de Santander, Cesar, Bolivar, Antioquia, and Cordoba. Matter of fact, these results are comparable with what is reported in the Humanitarian Needs Overview document, prepared in the name of the Humanitarian Country Team and OCHA, with the support of the Colombia Information Management and Analysis Unit (UMAIC in Spanish), and iMMAP Colombia (Humanitarian Country Team and OCHA, 2018). The areas highlighted in Figure 8b belong to departments that, in the document, show the highest number of people affected by natural disasters in the time window 2016–2018. We can observe almost the same pattern in Figure 8c related to the relationship between the illiteracy rate (proxy to education) and conflicts. We use the natural gas coverage as a proxy of access to the utility's service and the significant coefficients show the expected negative relationship (Figure 8d).

### 3.5. Analysis on a restricted study area

**Figure 9:**
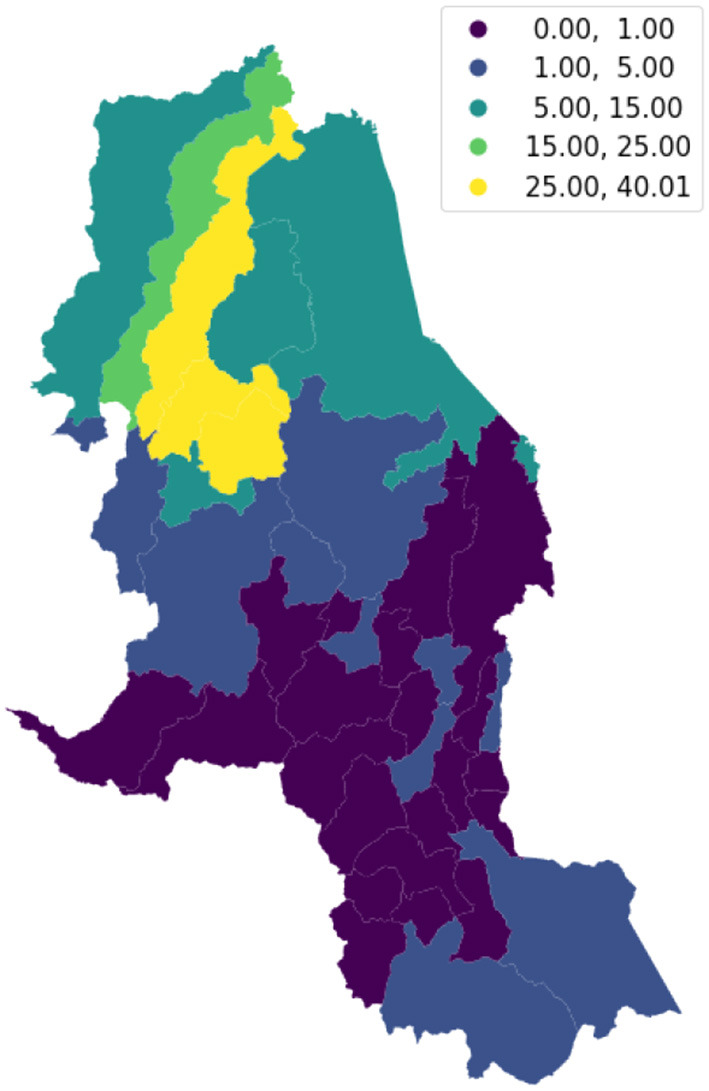
Distribution of violent events in 2018, per 10,000 inhabitants, per municipality of Norte de Santander department.

**Figure 10:**
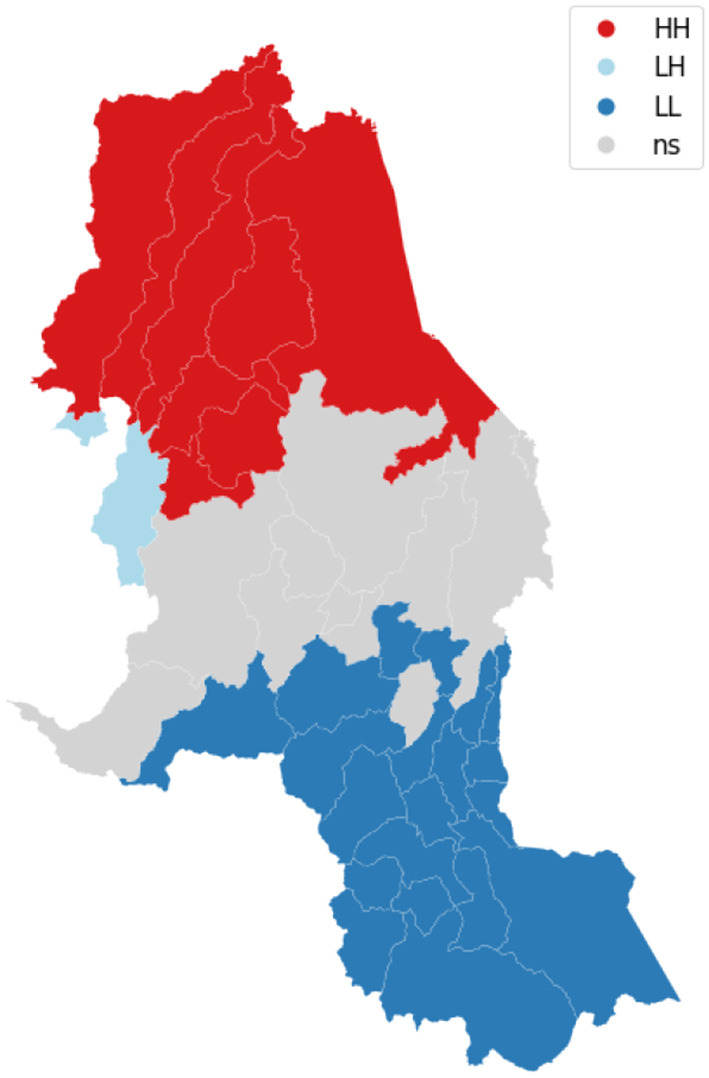
LISA clusters map—Focus on Norte de Santander.

Norte de Santander is one of 32 departments in Colombia and is known to be highly affected by violence and conflicts. According to the teams of Médecins Sans Frontières (MSF), 'People in the Norte de Santander and Nariño regions of Colombia are experiencing high levels of violence as a result of disputes between various armed groups, including mass killings, assassinations, threats and displacement from their homes' (Frontières, 2020). We focus on the department of Norte de Santander because it proves to be an interesting use case as one of the most recurrent significant areas pointed out by the exploratory local analysis through GWR. We try to understand the spatial effects on a restricted area and we apply the same pipeline: spatial analysis, analysis of determinants and spatial regression model. Figure 9 shows the concentration of violent events in 2018, by the municipality. Then, we test the hypothesis of nonzero spatial autocorrelation in violent events by performing Moran's statistics. The Moran's *I*-value is 0.51 and the related *p*-value is 0.001, so statistically significant. Thus, we reject the hypothesis of complete spatial randomness. We analyze the local spatial autocorrelation which describes similarities and dissimilarities of a municipality and its neighboring municipalities and we provide a statistic for each location by performing the LISA analysis (Figure 10). We select the final set of covariates by applying the above-mentioned feature selection procedure. Predictive variables that have proven to be the most frequent in the selection procedure and the most informative ones to explain conflicts are cocaine cultivation areas, droughts, indigenous reservations, natural disasters in 2017, and net coverage in basic education. Compared to the global analysis, if we focus on a restricted area we can see other exogenous variables emerging and be taken into account. Thus, we state that the global study hides some specific signals at a local scale. Again, the lag model proves to be more suitable to explain the conflicts phenomena than the error model. The Lagrange Multiplier tests results are reported in Table 3.

**Table 3:** Lagrange multiplier tests.

	**Spatial lag (H0 : ρ = 0)**	**Spatial error (H0 : λ = 0)**
LM	5.47 (0.019)	0.18 (0.669)
Robust LM	7.40 (0.006)	2.12 (0.15)

The numbers in parentheses are *p*-values. Analysis restricted to Norte de Santander department.

**Figure 11:**
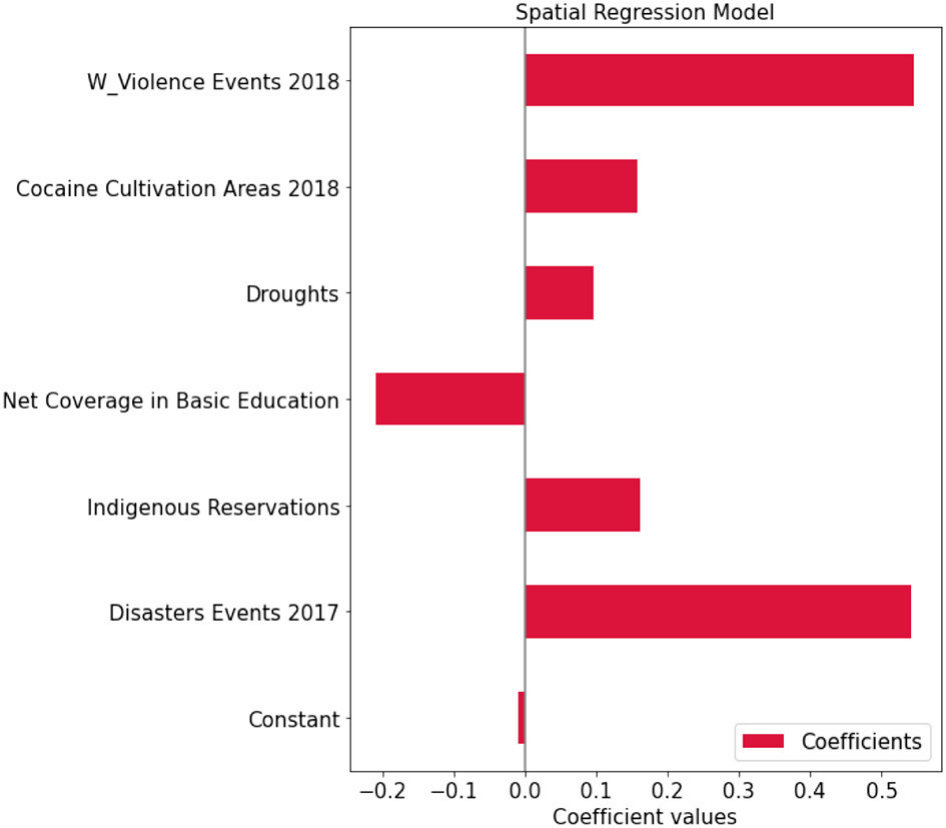
Regression coefficients—analysis on Norte de Santander.

We show results related to the lag model, in which the Pseudo R-squared is equal to 0.78 and the Spatial Pseudo R-squared is equal to 0.78. Results prove that natural disasters in 2017 and droughts are positively related to conflicts and have significance, net coverage in basic education is negatively related to conflicts and has significance, while indigenous reservation and cocaine cultivation areas seem to be not significant. The spatial lag term of conflicts (W ViolenceEvents 2018) has a positive effect and it is highly significant. Figure 11 shows the spatial regression coefficients.

## 4. Discussion

Conflict phenomena are a complex system. In this project, we explore particular aspects in terms of poor social conditions and environmental disasters that could trigger violent events and cause social vulnerability. More in detail, we run a spatial analysis to study whether the dependent variable y (conflict events) in place i is affected by the independent variables in both place i and j. Surprisingly, information related to a social condition such as access to public health and employment do not play a relevant role in any global spatial regression model tested. Economic features such as investments in agriculture, transportation, or total income show a very little relationship with conflict events, not even social ones. We find out that natural disasters, illiteracy rate and the percentage of cocaine areas are the most relevant exogenous variables. Notably, the sensitivity analysis proves that different feature selection approaches produce consistent results.

Focusing on the previous years conflict data, we conclude that the autoregressive component is the baseline and provides almost the total predictive power. The exogenous variables play a minor role, but they provide useful information, especially when explored locally, and improve the model. Thus, taking into account both components (autoregressive and exogenous data) is helpful to predict future conflicts pockets and to assess policy options that could tackle violent events. By identyfing the key drivers of violence in Colombia and which are the areas that are more suffering by conflicts, we have evidence to inform subnational governments and to support the decision making policies. Our results prove how natural disasters influence the increase in violent events. Therefore, programs are needed to deal with the effects of climate change. This can be done by strenghtening the social protection system that should include an institutional and policy framework that not only provides support rapidly to households affected by a crisis, but that also allows assessing and reducing the exposure of households to climate change risks before crises occur. The World Bank states that this could be achieved by integrating a Climate Change Vulnerability Index (like in the Dominican Republic) to social registries (Davalos, 2021). Well prepared social programs could ensure the resilience of the poorest to climate shocks, a rapid and flexible response to natural disasters that help in the decrease of mass displacement. This would, in turn, results in decrease in vulnerability and violence event. Discussing about education, we found relevant the illiteracy rate, especially in rural areas. It would be important planning an intensification of learning in basic competencies and making more equitable and flexible the access to the tertiary education. In addition to this, information such as electric coverage and gas coverage influence negatively the occurence of violence events. Thus, improving the infrastructure and services impacts in reducing inequalities across departments and municipalities. Regarding the cocaine areas, their relationship with conflicts have been proven and largely discuss, and we also have found the impact of this feature on the target variable. The drug war is one of the main challenging and discussed issues in Colombia. The gangs fight fiercely to control the cocaine trade and in some places such as remote parts of Colombia, they are rich, powerful and well armed. In this context, it has been proposed to legalize cocaine for home use. However, legalizing cocaine may not stop international crime and may allow gangs to dominate the new legal markets just as they have dominated the illegal ones (Economist, 2022). One of the critical points is that selling coca leaves is advantageous for local farmers because they find lots of buyers. Furthermore, the high demand for drugs stimulates both illicit production and trafficking. In this context, the problem requires corrective measures through governments policies and programs. An interesting example is the UN/Thai programme for drug abuse control which was carried out during the years 1972–1979. The purpose of the United Nations Programme for Drug Abuse Control in Thailand has been to find ways of reducing the illicit supply of opium through a pilot project of technical and social aid for crop substitution activities. Poppy growers were helped to grow crops other than the opium poppy: crops thay gave them an income equal to or better than the money they had received from the illicit sale of opium (Williams, 1979). Also in Colombia on January 2017 was carried out a similar peacebuilding policy: a new program to substitute crops used for illegal purposes with alternative livelihoods (PNIS). However, the PNIS has produced more, not less violence and coca plantations, as it was opposed violently by criminal groups. The communities welcomed the PNIS as a way out of a lucrative, but insecure and violent livelihood, but the alternative crops produced a lower income level than the coca leaves. Farmers returned to coca since provides economic stability (Nilsson and Marín, 2021). A test could be to try and suggest some successful crops to encourage farmers to switch from growing coca to other crops without incurring lower wages. Furthermore, reducing the demand for cocaine is a key point and could be achieved by promoting facilities for the treatment, rehabilitation, and social reintegration of drug addicts and developing educational materials and programs suitable for high-risk populations. However, those strategies take time to have a direct impact. Finally, violence is primarily the outcome of past violence: once a place experiences conflict events, it is significantly more likely to experience additional episodes of violence. To ensure that nations emerge from conflicts and violence the social ties must be rebuilt and victims and civilians must be supported. Therefore, extremly important is investing resources to organize frameworks that helps in breaking the cycle of violence through programs that brings individuals and communities together.

Comparing the spatial error model and the spatial lag model we notice that the most suitable spatial regression model to explain conflicts is the second one. Spatial lag suggests a possible diffusion process, thus events in one place predict an increased likelihood of similar events in neighboring places, that is consistent with what we expect. Starting from the assumption that what we can see globally is driven by what happens locally, we search which are the significant areas. The local exploration through GWR shows how the global phenomenon hides a strong localization at lower spatial units. In particular, despite some variables seeming to be the more informative to explain the process globally, they highlight a strong relationship for only a few specific areas when considering a local analysis. This result proves the importance of moving to research focused on a restricted study area. By considering a specific area (Norte de Santander), one of the most affected by vulnerability and violence, we prove that natural disasters and especially drought conditions are significant and should be taken into account. However, just a few socioeconomic variables are highly related to conflict events (in detail, coverage in basic education). Narrowing down the study area brings with its pro and cons: on the one hand, we can detect whether there is some interesting and functional information for a certain area that does not appear while studying a larger territory; on the other hand, we have to face with the decrease in the number of observation points. Exploring the relationship among environmental, socioeconomic, and conflict-related data contributes to improving knowledge of a very complex system and brings several challenges ahead for constructive future studies. First, there are lots of other countries affected by conflicts on which nonprofit organizations need to make informed decisions. Second, there are lots of other drivers to be taken into account: food insecurity is a big issue while talking about people's vulnerability and poor social conditions and it could lead to violence; people displacement from and to a neighboring country (e.g., Venezuela) is a relevant point to be investigated. Finally, a time series analysis could help in exploring the trend of conflicts across municipalities through time, and which shocks (climate shocks, economic shocks) lead to increase of violence.

## Data availability statement

Publicly available datasets were analyzed in this study. This data can be found at: https://terridata.dnp.gov.co/; https://monitor.salahumanitaria.co; https://datos.gov.co/; https://drive.google.com/drive/folders/1tiezF5erETYpdj0_XYaUA2QRuqG2g2uS?usp=sharing.

## Author contributions

DP, RS, CC, and SF conceived and planned the experiments. SF carried out the experiments and the data analysis and took the lead in writing the manuscript. DP, RS, and CC contributed to the interpretation of the results. All authors provided critical feedback and helped shape the research, analysis, and manuscript.
