# Supplementary material for: Combining environmental and socioeconomic data to understand determinants of conflicts in Colombia
Source: Front Big Data. 2023 Feb 16;6:1107785. doi: 10.3389/fdata.2023.1107785 (PMC9978383; doi:10.3389/fdata.2023.1107785)
Supplement: Supplementary file 1 [file Presentation_1.PDF]

Supplementary Material

1 GENERAL STATISTICS ON CONFLICT DATA

In this study, we focus on conflicts event in Colombia and we rely on an event-based dataset that includes nearly 3000 events over 400 colombian municipalities. We perform the spatial analysis at municipalities level (admin2 administrative unit) and we visualize the trend of conflicts by department level (admin1 administrative unit) in 2018. Figure S1 shows the number of events that occured per department after having normalized the number of conflicts of each muncipality for the population number. We highlight Norte de Santander, Narino and Antioquia as the most affected departments. Figure S2 shows the trend of conflicts per each month of the year 2018, comparing all departments. We observe heterogeneity among departments in terms of periods with increased or decreased conflicts. Also, we can found spikes in correspondance to a specific month, as for April 2018 in Norte de Santander.

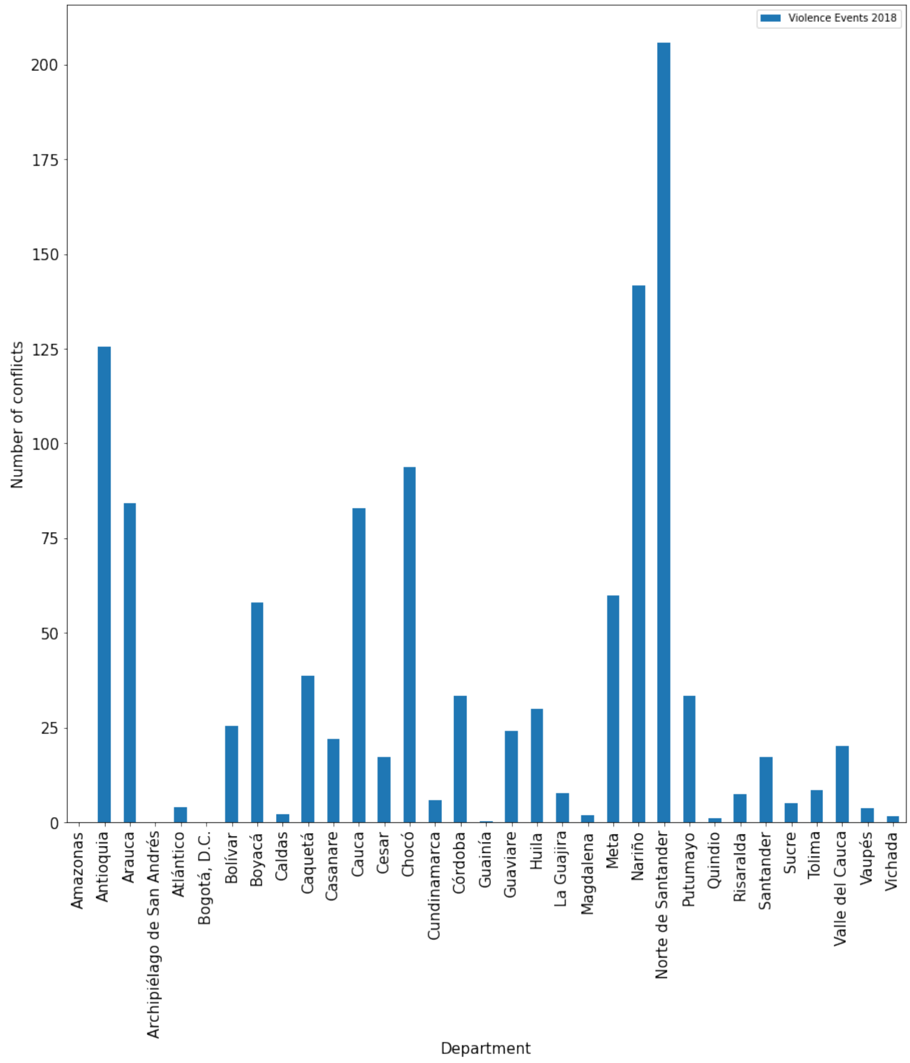

Figure S1. Number of events occured in 2018 per colombian department

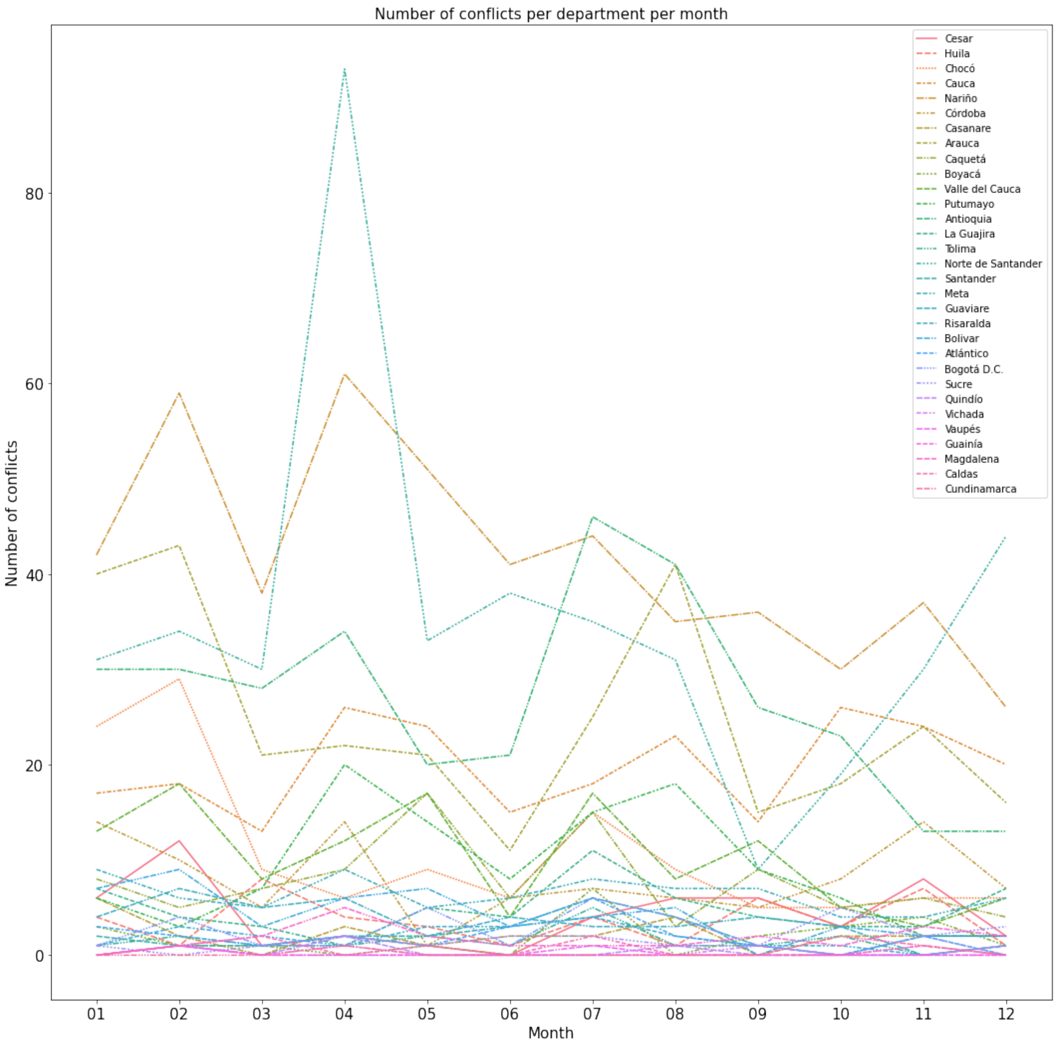

Figure S2. Number of events occured in 2018 per colombian department

2 DATA COLLECTION

In this project, we rely on open data. Regarding conflicts and disaster events we make use of the tool provided by the United Nations Office for Coordination of Human Affairs (OCHA) that allows monitoring of the Colombian situation per year and per different admin levels. Regarding social, economic, and specific environmental information, we use data from the Terridata platform, a tool created by the Colombian National Planning Department (DNP). A description of the 53 control variables and of the target variable can be found in Table S1 and Table S2.

Variable	Type	Source
Conflicts		
Violence events	number events per a thousand people	OCHA
Demography		
Indigenous population	people	TerriData, with Departamento Administrativo Nacional de Estadística (DANE) data from the National Population and Housing Census
Black, mulatto or Afro-Colombian population	people	TerriData, with DANE data
Raizal population	people	TerriData, with DANE data
Rom population	people	TerriData, with DANE data
Rural population	people	TerriData, with DANE data
Urban population	people	TerriData, with DANE data
Total ethnic population	people	TerriData, with DANE data
Total men	people	DANE
Total women	people	DANE
0-4 years	people	DANE
5-9 years	people	DANE
10-14 years	people	DANE
15-19 years	people	DANE
20-24 years	people	DANE
25-29 years	people	DANE
30-34 years	people	DANE
35-39 years	people	DANE
40-44 years	people	DANE
45-49 years	people	DANE
50-54 years	people	DANE
55-59 years	people	DANE
60-64 years	people	DANE
65-69 years	people	DANE
70-74 years	people	DANE
75-79 years	people	DANE
80-84 years	people	DANE
over 85 years	people	DANE
Education		
Gross coverage in basic education	%	TerriData, with Departamento Administrativo Nacional de Estadística (DANE) data from the 2018 Census
Net coverage in basic education	%	TerriData, with DANE data
Illiteracy Rate (Census)	%	TerriData, with DANE data
Rural Illiteracy Rate (Census)	%	TerriData, with DANE data
Urban Illiteracy Rate (Census)	%	TerriData, with DANE data

Table S1. Description of explanatory variables and data source - Table 1

Variable	Type	Source
Environment		
Disasters Events 2017	number events per a thousand people	OCHA
Disasters Events 2018	number events per a thousand people	OCHA
Mass Movement	eventos	TerriData, with DNP data from Unidad Nacional para la Gestión del Riesgo de Desastres (UNGRD)
Floods	eventos	TerriData, with DNP data from UNGRD
Droughts	eventos	TerriData, with DNP data from UNGRD
Earthquakes	eventos	TerriData, with DNP data from UNGRD
Forest Fires	eventos	TerriData, with DNP data from UNGRD
Cumulative total of disaster events	eventos	TerriData, with DNP data from UNGRD
Disaster Management	thousands of constant pesos	TerriData, with DNP data from FUT
Total in disaster management	thousands of constant pesos	TerriData, with DNP data from Formulario Único Territorial (FUT)
Finance		
Total Expenses	millions of current pesos	TerriData, with DNP data from FUT Formulario Único Territorial
Total Income	millions of current pesos	TerriData, with DNP data from FUT
Total Income Per Capita	millions of current pesos	TerriData, with DNP data from FUT and DANE
Investment - Agriculture	millions of current pesos	TerriData, with data from FUT
Investment - Environment	millions of current pesos	TerriData, with data from FUT
Investment - Social Promotion	millions of current pesos	TerriData, with data from FUT
Investment - Culture	millions of current pesos	TerriData, with data from FUT
Investment - Community Development	millions of current pesos	TerriData, with data from FUT
Investment - Education	millions of current pesos	TerriData, with data from FUT
Investment - Justice and Security	millions of current pesos	TerriData, with data from FUT
Investment - Disaster prevention and Care	millions of current pesos	TerriData, with data from FUT
Investment - Health	millions of current pesos	TerriData, with data from FUT
Investment - Transportation	millions of current pesos	TerriData, with data from FUT
Education	%	TerriData, with DNP data from FUT and Departamento Nacional de Planeación (SICODIS)
Health	%	TerriData, with DNP data from FUT and SICODIS
Economy		
Value added by economic activities Primary activities	billions of current pesos	TerriData, with DNP data from DANE
Value added by economic activities Secondary activities	billions of current pesos	TerriData, with DNP data from DANE
Access to public services		
Electric Power Coverage (Census)	%	TerriData, with DANE data
Natural Gas Coverage (Census)	%	TerriData, with DANE data
Internet Coverage (Census)	%	TerriData, with DANE data
Aqueduct Coverage (Census)	%	TerriData, with DANE data
Sewer Coverage (Census)	%	TerriData, with DANE data
Qualitative Housing Deficit (Census)	%	TerriData, with DANE data
Quantitative Housing Deficit (Census)	%	TerriData, with DANE data

Table S2. Description of explanatory variables and data source - Table 2

Variable	Type	Source
Health		
Mortality Rate	cases for 1000 inhabitants	TerriData, with Ministry of Health and Social Protection data (MSPS)
HIV Incidence	cases for 100000 inhabitants	TerriData, with MSPS data
Poor unserved population	people	TerriData, with MSPS data
Job Market		
Percentage of formally employed persons	%	TerriData, with DNP data from FILCO - Ministry of Labour
Companies Generating Formal Employment	number of companies	TerriDatawith DNP data from FILCO
Territorial Planning		
Indigenous reservation	hectares	TerriData, with DNP data from Instituto Geográfico Agustín Codazzi (IGAC)
Cocaine Cultivation Areas	% per municipality	GOVCO portal
Coffee Areas - Sown Areas	hectares	GOVCO portal
Coffee Areas - Harvested Areas	hectares	GOVCO portal
Coffee Production	tons	GOVCO portal
Coffee Yield	tons/hectares	GOVCO portal

Table S3. Description of explanatory variables and data source - Table 3

3 ANALYSIS OF DETERMINANTS

3.1 Multicollinearity Analysis

The first step of filtering is focused on avoiding redundancy. We check for multicollinearity and thus if we observe variables with a correlation coefficient among each other higher than 0.8, we drop out from the set of features the one that shows a weaker relationship with the target. After this processing, we filter out the features shown in Table S4.

**Variables**
Cumulative total of disaster events
Education
Gross Coverage in Basic Education
Health
Illiteracy Rate (Census)
Investment - Attention to vulnerable groups - social promotion
Investment - Community Development indigenous reservations
Investment - Culture
Investment - Education
Investment - Health
Investment - Justice and security
Investment - Prevention and attention to disasters
Investment - Transportation
Rom population
Total ethnic population
Total expenses
Total in disaster management
Total income
Total men
Total women
0-4 years
5-9 years
10-14 years
15-19 years
20-24 years
25-29 years
30-34 years
35-39 years
40-44 years
45-49 years
50-54 years
55-59 years
60-64 years
65-69 years
70-74 years
75-79 years
80-84 years
Coffee Areas - Sawn Areas
Unattended poor population - PPNA
Urban population
Value added by economic activities - Secondary activities

Table S4. Explanatory variables filtered out after the first *feature selection* step

3.2 Feature Selection

Then, we implement the Forward Stepwise selection strategy to reduce model complexity. We take into account the stability of random perturbations of training samples by randomly subsampling for 300 times the 63.2% of the initial dataset and running the selection procedure on the subset, considering the Akaike's Information Criteria (AIC) minimum value. We select features that occur at least in 150 tests. Figure S3 shows the results, Table S5 reports the final subset of features selected and Figure S4 shows variables distribution.

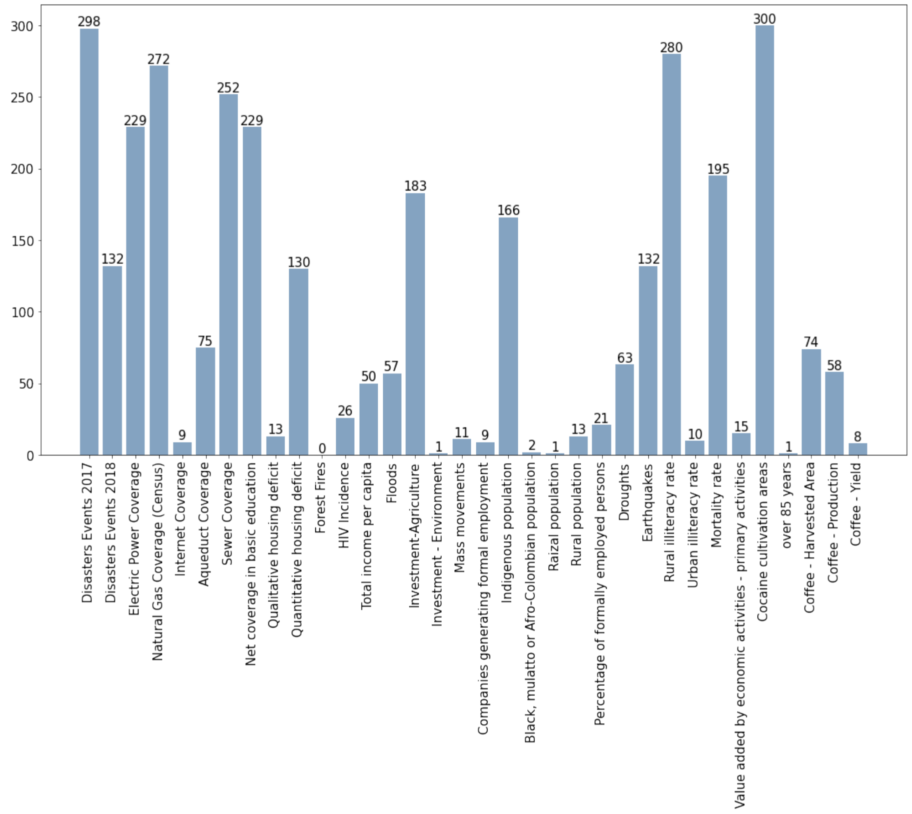

Figure S3. Occurence of each variable in the analysis of determinants

**Variables**
Cocaine Cultivation Areas
Disasters Events 2017
Electric Power Coverage
Indigenous Population
Investment - Agriculture
Mortality Rate
Natural Gas Coverage
Net Coverage in Basic Education
Rural Illiteracy Rate
Sewerage coverage

Table S5. Explanatory variables considered after *feature selection* step

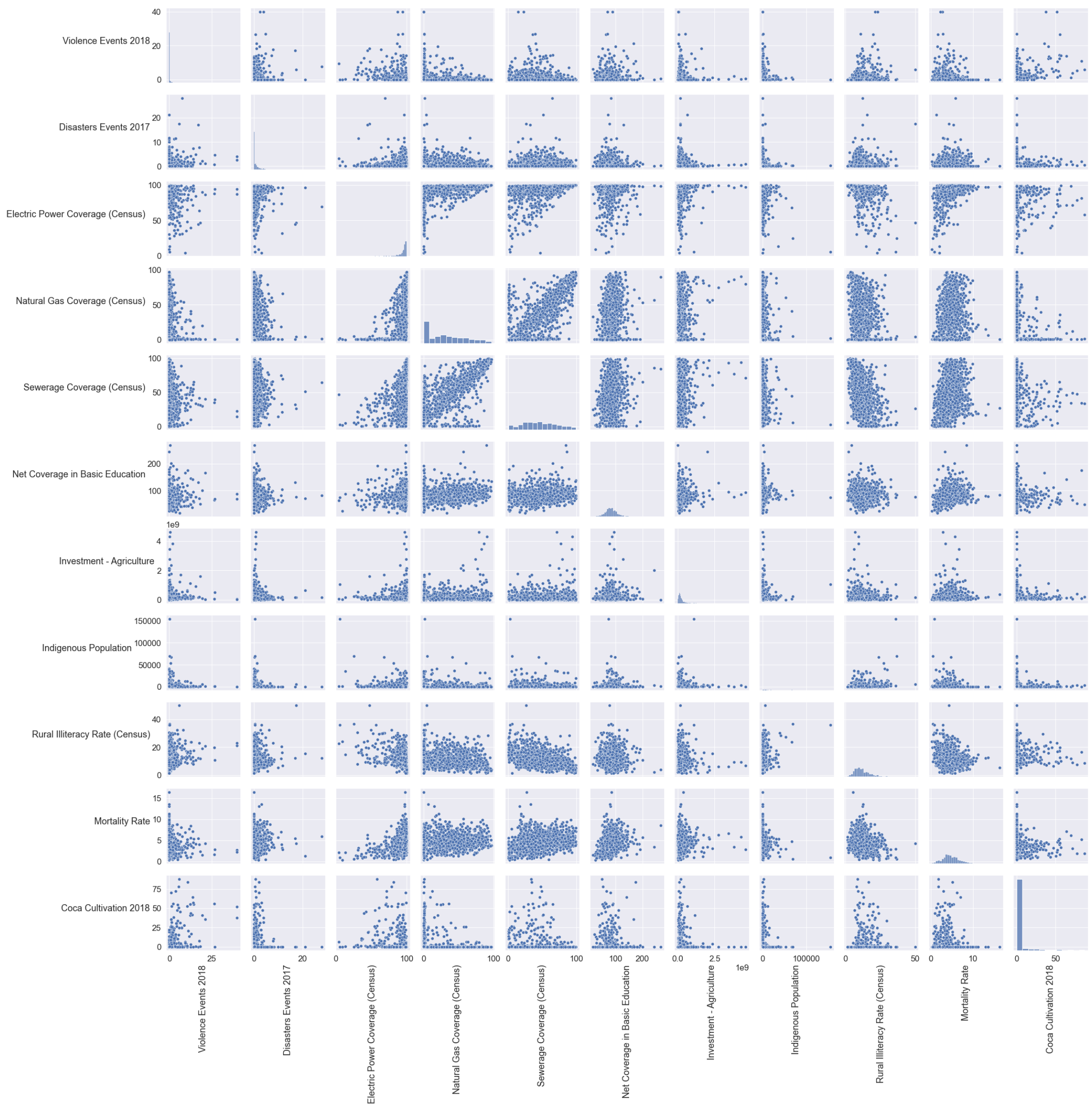

Figure S4. Distribution of selected variables

We have also analyzed the outcome of two additional feature selection approaches on the variables final set: VIF (variance inflation factor) method and Random Forest method based on Gini.
With respect to the VIF method, as already done for the first approach, we reduce the number of features from 75 to nearly 40 by looking at multicollinearity through the Pearson correlation coefficient. Then, we compute the VIF factor: the results are reported in Table: S6. A rule of thumb is that if $VIF > 10$, then multicollinearity is high. For this reason, we repeatedly remove all the features with VIF factor higher than 10. The Table shows the final set of covariates.
As regards the Random Forest method based on Gini, we select the most important fifteen features as final set of covariates: Figure S5 shows the feature importance.
In the section 5 we discuss about the two different feature selection approaches that eventually produce similar results.

Feature	VIF	Feature	VIF
Floods	1095.91	Investment - Agriculture	1.98
Droughts	2.95	Earthquakes	3.39
Electric Power Coverage	71.08	Investment - Environment	7.16
Natural Gas Coverage	6.52	Investment - Prevention to disasters	7.67
Illiteracy Rate (Census)	53.11	Rural Illiteracy Rate (Census)	30.61
Internet Coverage	7.67	Mass Movements	185.89
Aqueduct Coverage (Census)	28.73	Companies Generating Formal Employment	3.42
Urban Illiteracy Rate (Census)	15.62	Mortality Rate	10.46
Sewerage Coverage	15.64	Indigenous Population	8784.31
Net coverage in basic education	12.61	Black, mulatto or Afro-Colombian population	32383.15
Cumulative total of disaster events	4686.84	Value added by economic activities - Primary	1.35
Qualitative housing deficit	17.51	Raizal population	4.86
Quantitative housing deficit	4.67	Rom population	3.34
Disaster Events 2017	1.59	Disaster Events 2018	1.42
Forest Fires	1172.86	Rural Population	4.59
Incidence VIH-ODS	1.39	Total ethnic population	45415.14
Cocaine Cultivation Areas	1.33	Over 85 years	14.9
Total income per capita	4.04	Percentage of formally employed persons	1.19
Coffee - Sown Area	5.45	Coffee - Harvested Area	5.46
Coffee - Production	3.41	Coffee - Yield	2.90

Table S6. VIF table

Feature	VIF	Feature	VIF
Natural Gas Coverage	4.67	Internet Coverage	5.84
Net coverage in basic education	9.17	Quantitative housing deficit	2.77
Incidence VIH-ODS	1.32	Total income per capita	3.61
Investment - Agriculture	1.83	Investment - Environment	4.93
Investment - Prevention to disasters	4.35	Companies Generating Formal Employment	3.17
Raizal population	1.88	Rom population	1.84
Percentage of formally employed persons	1.17	Droughts	1.30
Earthquakes	1.26	Rural Illiteracy rate	9.05
Urban Illiteracy Rate	8.08	Value added by economic activities - Primary	1.26
Disasters Events 2017	1.44	Disasters Events 2018	1.38
Coca Cultivation Areas	1.27	Coffee - Sown Area	5.40
Coffee - Harvested Area	5.40	Coffee - Production	3.39
Coffee - Yield	2.28		

Table S7. VIF table - final set of covariates

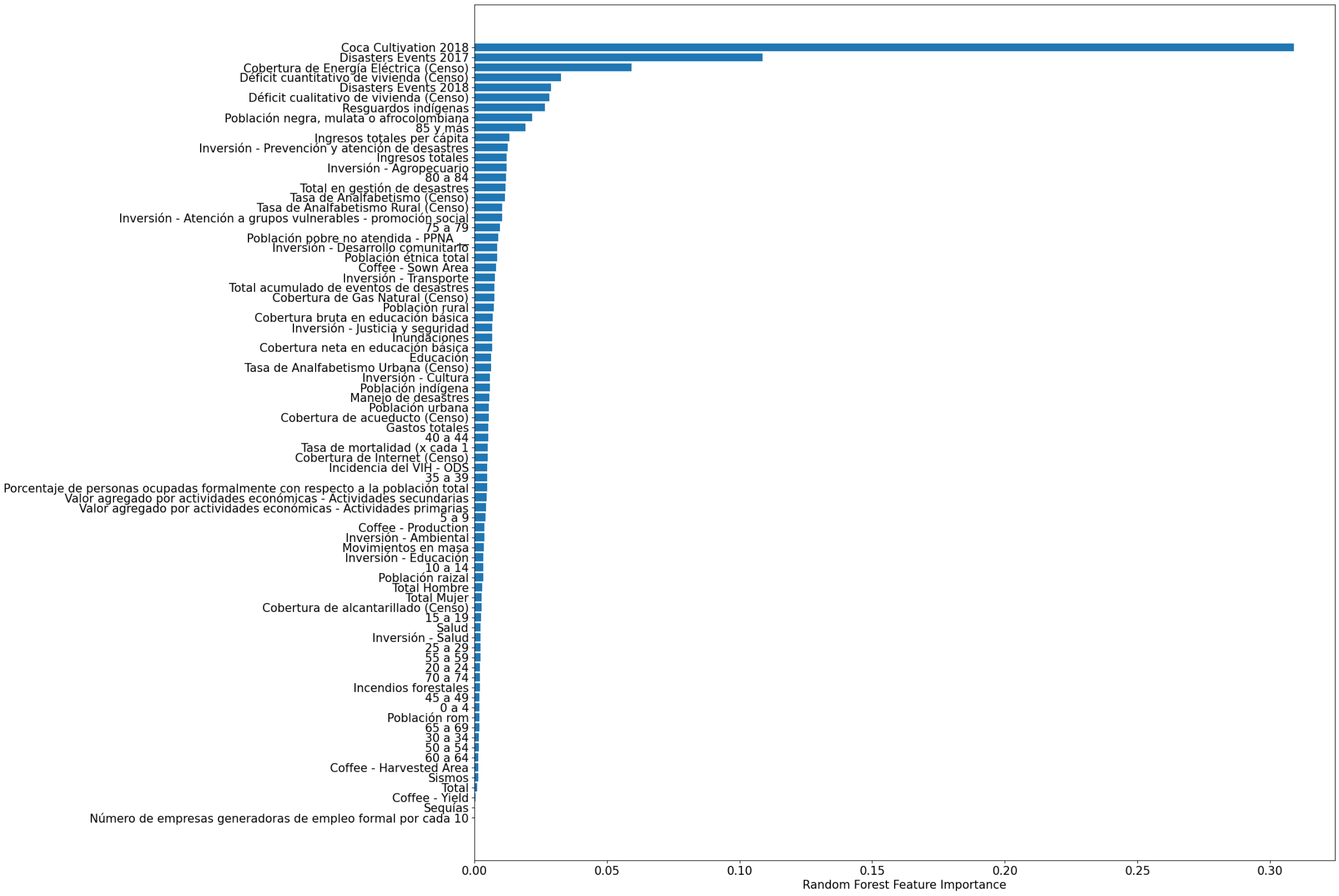

Figure S5. Random Forest based on Gini - Feature importance

4 SPATIAL EXPLORATION

We test the spatial autocorrelation of the conflicts-related process through the Moran's I statistics. The Moran scatterplot is an illustration of the relationship between the values of the attribute (conflicts) at each location and the average value of the same attribute at neighboring locations. Through the local analysis, we are able to distinguish which are areas with lower or higher spatial correlations. In particular, areas are divided in:

- High-High: high values surrounded by high values;
- Low-Low: low values surrounded by low values;
- High-Low: high values surrounded by low values;
- Low-High: low values surrounded by high values;
- ns: non significant.

Figure S6 and Figure S7 show respectively the Global and Local Moran's I scatterplot.

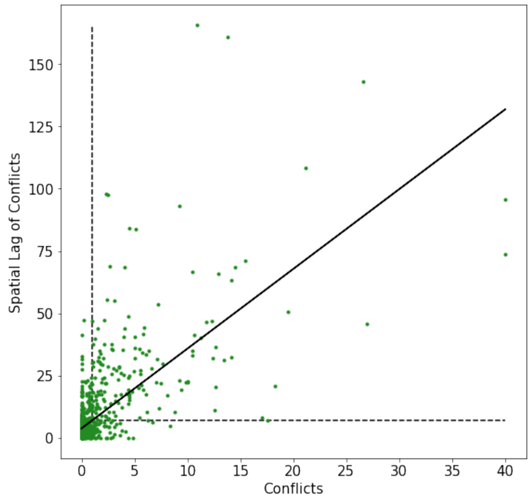

Figure S6. Global Moran Scatterplot

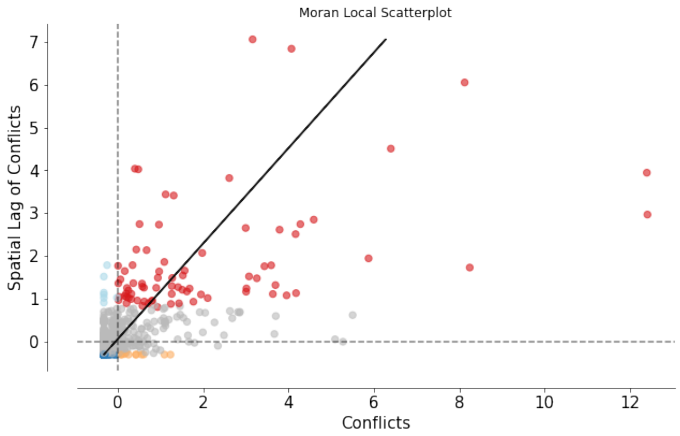

Figure S7. Local Moran Scatterplot

5 GLOBAL SPATIAL REGRESSION MODELS

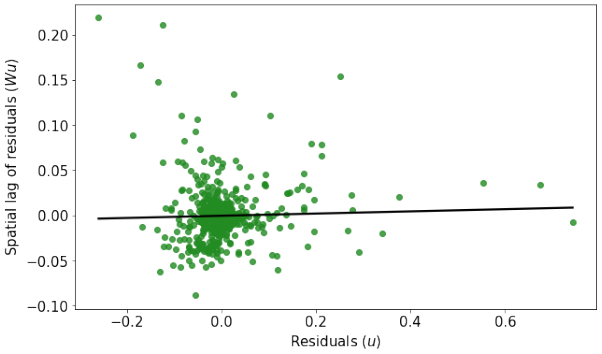

Figure S8. Residuals versus spatial lag of residuals - Spatial Lag Model

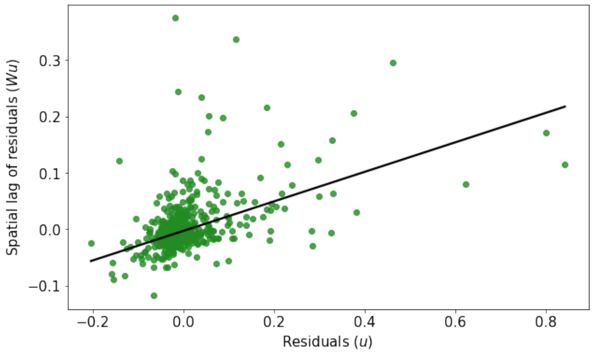

Figure S9. Residuals versus spatial lag of residuals - Spatial Error Model

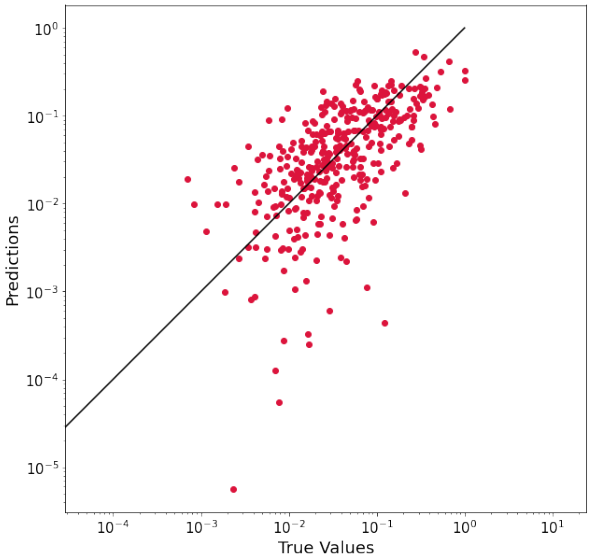

Figure S10. Actual versus predicted value - Spatial Lag Model

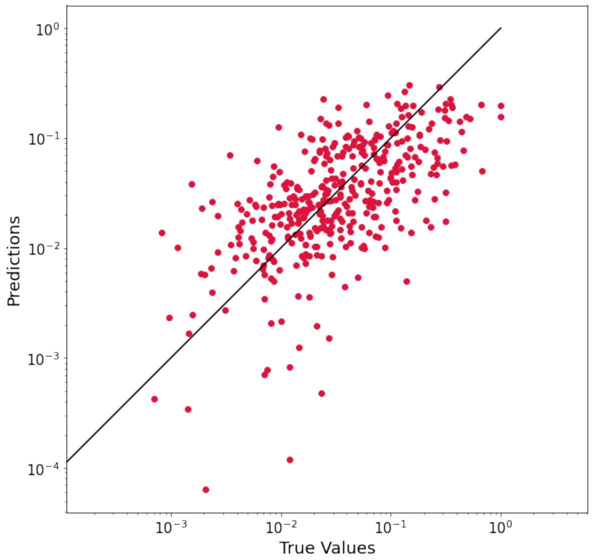

Figure S11. Actual versus predicted value - Spatial Error Model

We test the regression model by using the variables final set selected by using the two additional feature selection approaches.

With respect to the VIF method, we run both the Lagrange Multiplier test and the spatial models with the new subset of features reported in Table S7. The general results do not change because the spatial lag model turns out to be more suitable than the spatial error model, and the features that show significant p-values are always the same: cocaine cultivation areas, natural disasters in 2017, rural illiteracy rate. However, in this case, the electric power coverage is filter our from the subset of feature and the natural gas coverage shows a p-value higher than 0.05. By considering less features in the model, with our subset of features, the electric power coverage and the natural gas coverage shows a negative correlation with conflicts with p-values lower than 0.05. Table S8 shows the regression summary.

As regards the Random Forest based on Gini method, we run again the Lagrange Multiplier test and the spatial models and we obtain similar results. Also in this case, the set of features that show significant

p-values are: cocaine cultivation areas, natural disasters in 2017, rural illiteracy rate and electric power coverage. In this case, the natural gas coverage variable get filtered out in the feature selection process. Table S9 shows the regression summary.

Summary of Output Spatial Lag Model

Pseudo R-squared = 0.49
Spatial Pseudo R-squared = 0.34
Sigma-square ML = 0.003
S.E of regression = 0.057
Log likelihood = 1568.41
Akaike info criterion = -3080.83
Schwarz criterion = -2940.71

**Variable**	**Coefficient**	**Std. Error**	**z-Statistic**	**Probability**
CONSTANT	-0.019	0.008	-2.378	0.017
Natural Gas Coverage	-0.014	0.009	-1.602	0.109
Internet Coverage	0.017	0.019	0.856	0.392
Net Coverage in Basic Education	0.027	0.020	1.322	0.186
Quantitative Housing Deficit	0.023	0.011	2.067	0.038
Incidence VIH-ODS	0.022	0.045	0.490	0.623
Total income per capita	0.005	0.026	0.202	0.839
Investment - Agriculture	0.042	0.025	1.663	0.096
Investment - Environment	-0.002	0.086	-0.026	0.979
Investment - Prevention to disasters	-0.001	0.063	-0.014	0.989
Companies Generating Formal Employment	-0.030	0.047	-0.658	0.511
Raizal population	0.008	0.074	0.109	0.913
Rom population	0.008	0.063	0.130	0.896
Rural population	-0.017	0.026	-0.673	0.501
Percentage of formally employed persons	-0.015	0.048	-0.318	0.750
Droughts	-0.009	0.016	-0.613	0.539
Earthquakes	0.010	0.015	0.687	0.492
Rural Illiteracy Rate	0.046	0.021	2.174	0.029
Urban Illiteracy Rate	-0.010	0.018	-0.567	0.571
Value added by economic activities	-0.047	0.042	-1.120	0.263
Disasters Events 2017	0.15	0.026	5.729	0.000
Disasters Events 2018	0.021	0.017	1.229	0.219
Coca Cultivation Areas	0.198	0.018	10.897	0.000
Coffee - Sown Areas	0.022	0.016	1.386	0.166
Coffee - Harvested Areas	-0.007	0.016	-0.406	0.685
Coffee - Production	-0.006	0.013	-0.479	0.632
Coffee - Yield	0.001	0.006	0.224	0.822
W Violence Events 2018	0.616	0.033	18.551	0.000

Table S8. Summary of output Spatial Lag Model - VIF method for feature selection

Summary of Output Spatial Lag Model

Pseudo R-squared = 0.48
Spatial Pseudo R-squared = 0.34
Sigma-square ML = 0.003
S.E of regression = 0.057
Log likelihood = 1564.26
Akaike info criterion = -3094.52
Schwarz criterion = -3009.45

Variable	Coefficient	Std. Error	z-Statistic	Probability
CONSTANT	0.032	0.017	1.898	0.058
Qualitative Housing Deficit	-0.001	0.009	-0.103	0.918
Total income per capita	0.009	0.023	0.378	0.706
Total income	0.051	0.474	0.107	0.914
Investment - Agriculture	0.035	0.024	1.418	0.156
Investment - Prevention to disasters	0.009	0.108	0.091	0.927
Rural Illiteracy Rate	0.037	0.017	2.157	0.031
Disasters Events 2017	0.141	0.026	5.501	0.000
Disasters Events 2018	0.024	0.017	1.398	0.162
Coca Cultivation Areas	0.199	0.018	11.138	0.000
Electric Power Coverage	-0.045	0.017	-2.706	0.007
Indigenous Reservation	-0.062	0.045	-1.358	0.174
80 - 84 years old	-0.318	1.405	-0.226	0.821
Over 85 years old	0.183	1.446	0.127	0.899
Total disaster management	0.072	0.322	0.223	0.824
W Violence Events 2018	0.618	0.033	18.833	0.000

Table S9. Summary of output Spatial Lag Model - Random Forest based on Gini method for feature selection

6 EXPLORING PAST CONFLICTS

We have available data about the number of conflicts per municipality from 2008 to 2018. Since our main goal is to run a spatial model considering observations of all the Colombian municipalities, we explore the correlation between the number of conflicts in 2018 and all the previous years. Figure S12 reports the heatmap with all the correlation coefficients. To avoid multicollinearity problems and redundancy, we filter out all the features that show a coefficient among each other higher than 0.8. Finally, we select the feature that present the strong correlation with the outcome variable: in Figure S13 we can see that the variable related to conflicts in 2017 has the highest coefficient. In Table S10 we report the regression coefficients of the spatial regression model, while Figure S14 and Figure S15 show, respectively, the residuals versus the spatial lag of residuals plot and the actual versus predicted values plot.

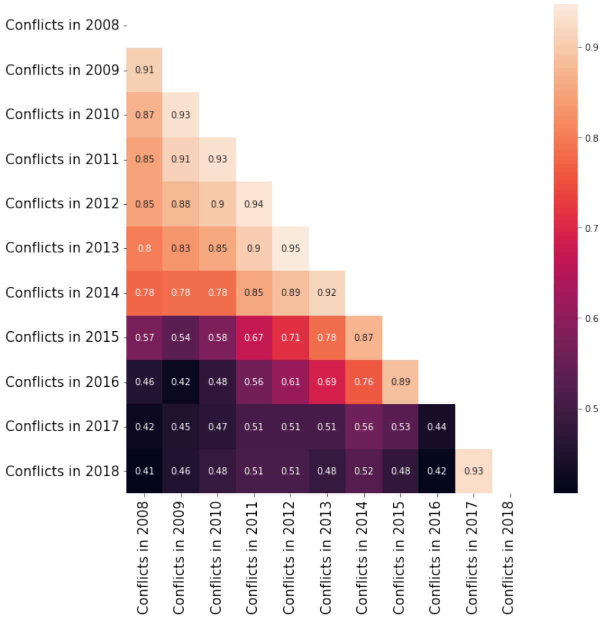

Figure S12. Correlation coefficient heatmap

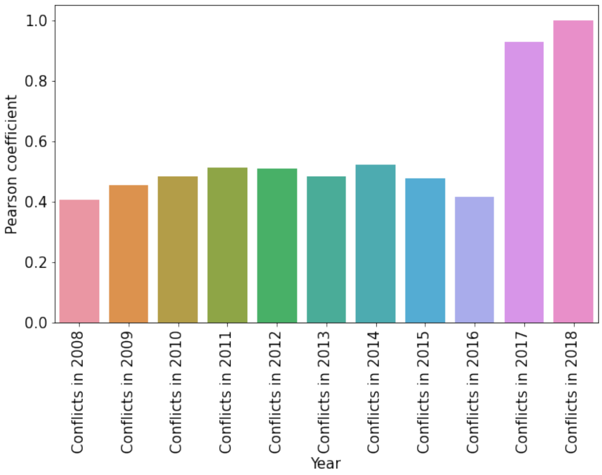

Figure S13. Barplot showing the correlation between each independent variable and the outcome variable

Summary of Output Spatial Lag Model

Pseudo R-squared = 0.61
Spatial Pseudo R-squared = 0.55
Sigma-square ML = 0.002
S.E of regression = 0.050
Log likelihood = 1728.83
Akaike info criterion = -3431.65
Schwarz criterion = -3366.60

Variable	Coefficient	Std. Error	z-Statistic	Probability
CONSTANT	-0.070	0.016	-4.432	0.000
Coca Cultivation Areas	0.125	0.016	7.626	0.000
Disasters Events 2017	0.087	0.022	3.922	0.000
Electric Power Coverage	0.056	0.016	3.701	0.000
Indigenous Population	0.021	0.035	0.583	0.560
Investment - Agriculture	0.019	0.020	0.948	0.343
Mortality Rate	-0.020	0.016	-1.186	0.236
Natural Gas Coverage	-0.006	0.007	-0.834	0.404
Net Coverage in Basic Education	0.041	0.016	2.490	0.013
Rural Illiteracy Rate	0.027	0.015	1.764	0.078
Sewer Coverage	-0.002	0.009	-0.223	0.823
Violence Events 2017	0.630	0.032	19.384	0.000
W Violence Events 2018	0.061	0.004	13.955	0.000

Table S10. Summary of output Spatial Lag Model

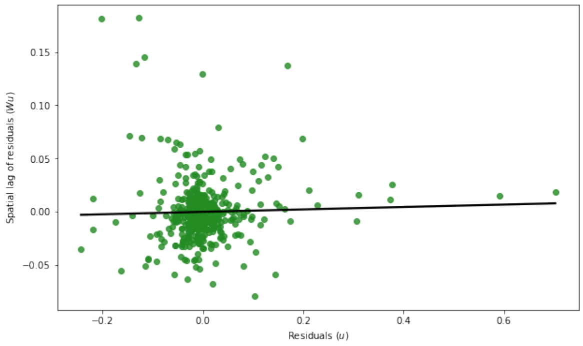

Figure S14. Adding past conflicts information: Residuals versus spatial lag of residuals - Spatial Lag Model

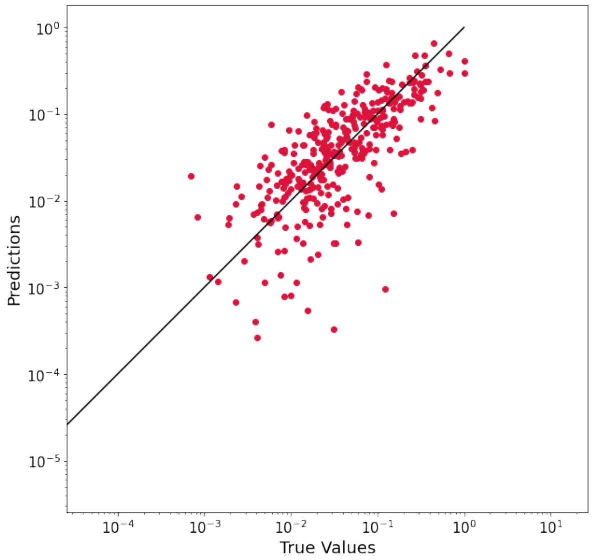

Figure S15. Adding past conflicts information: Actual versus predicted values - Spatial Lag Model

7 ANALYSIS ON A RESTRICTED AREA

	Spatial lag (H0 : $\rho = 0$)	Spatial error (H0 : $\lambda = 0$)
LM	5.47 (0.019)	0.18 (0.669)
Robust LM	7.40 (0.007)	2.12 (0.145)

Table S11. Norte de Santander department: Lagrange Multiplier Tests. (The numbers in parentheses are p-values).

Summary of Output Spatial Lag Model

Pseudo R-squared = 0.78
Spatial Pseudo R-squared = 0.78
Sigma-square ML = 0.013
S.E of regression = 0.116
Log likelihood = 28.68
Akaike info criterion = -43.35
Schwarz criterion = -31.53

Variable	Coefficient	Std. Error	z-Statistic	Probability
CONSTANT	-0.009	0.038	-0.243	0.808
Coca Cultivation Areas	0.157	0.116	1.348	0.178
Disasters Events 2017	0.541	0.100	5.391	0.000
Indigenous reservation	0.161	0.098	1.648	0.099
Net Coverage in Basic Education	-0.208	0.098	-2.120	0.034
Droughts	0.097	0.048	2.010	0.044
W Violence Events 2018	0.546	0.154	3.541	0.000

Table S12. Norte de Santander department: Summary of output Spatial Lag Model

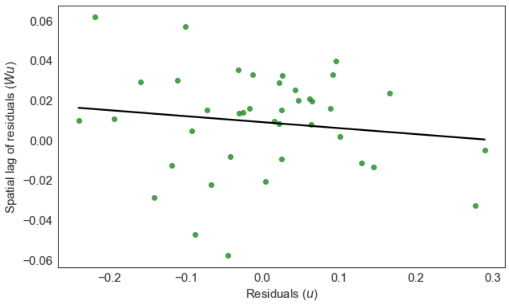

Figure S16. Norte de Santander department: Residuals versus spatial lag of residuals - Spatial Lag Model

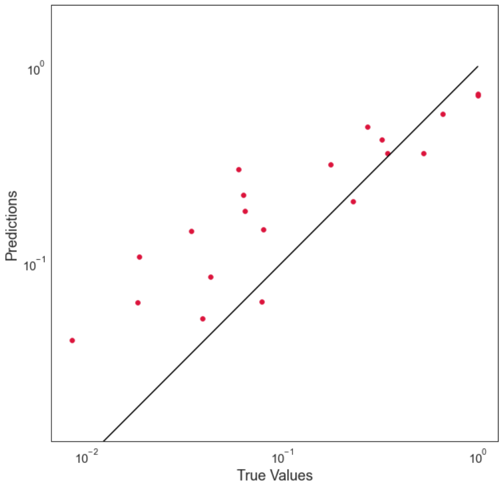

Figure S17. Norte de Santander department: Actual versus predicted values - Spatial Lag Model
